# Structure- and Ligand-Based Virtual Screening for Identification of Novel TRPV4 Antagonists

**DOI:** 10.3390/molecules30010100

**Published:** 2024-12-30

**Authors:** Atefeh Saadabadi, Linda Wilkman, Marja Rantanen, Ari-Pekka Koivisto, Outi M. H. Salo-Ahen

**Affiliations:** 1 Structural Bioinformatics Laboratory, Faculty of Science and Engineering, Åbo Akademi University, Tykistökatu 6, 20520 Turku, Finland; 2 Pharmaceutical Sciences Laboratory, Faculty of Science and Engineering, Åbo Akademi University, Tykistökatu 6, 20520 Turku, Finland; 3 Laboratory of Molecular Science and Engineering, Faculty of Science and Engineering, Åbo Akademi University, Henrikinkatu 2, 20500 Turku, Finland; 4 Pain Therapy Area, Orion Pharma, Tengströminkatu 8, 20360 Turku, Finland; marja.rantanen@orionpharma.com (M.R.); ari-pekka.koivisto@orionpharma.com (A.-P.K.)

**Keywords:** TRPV4 antagonists, molecular docking, MD simulation, pharmacophore modeling, FLIPR assay

## Abstract

Transient receptor potential vanilloid (TRPV) 4 is involved in signaling pathways specifically mediating pain and inflammation, making it a promising target for the treatment of various painful and inflammatory conditions. However, only one drug candidate targeting TRPV4 has entered the clinical trials. To identify potential TRPV4 inhibitors for drug development, we screened a library of ion channel-modulating compounds using both structure- and ligand-based virtual screening approaches. Since a high-resolution experimental structure of the human TRPV4 (hTRPV4) was not available during this study, we used a comparative model of hTRPV4 for the structure-based screening by molecular docking. The ligand-based virtual screening was performed using the pharmacophoric features of two known TRPV4 antagonists. Five potential hits were selected based on either the binding stability or the pharmacophore match, and their effect on hTRPV4 was tested using a FLIPR^tetra^ assay. All tested compounds inhibited hTRPV4 at 30 µM, with compound Z1213735368 showing an IC_50_ of 8 µM at a concentration of 10 µM. Furthermore, natural stilbenoids, known to modulate other TRP channels, were evaluated for their hTRPV4 binding and inhibitory potential. The findings provide insight into the structural determinants of hTRPV4 modulation and may facilitate further efforts in developing therapeutic hTRPV4 ligands.

## 1. Introduction

Transient receptor potential vanilloid 4 (TRPV4) is a non-selective cation channel that is a member of the vanilloid TRP subfamily (TRPV1–6) and is one of the thermosensitive TRPVs (thermoTRPVs, V1–V4). This channel is broadly expressed in multiple tissues and plays an important role in many physiological processes, specifically inflammation and nociception. For example, it is expressed in sensory neurons (in dorsal root ganglion and trigeminal ganglion) [1] and in many non-neuronal cells (e.g., skin keratinocytes, osteoclasts, fibroblasts, central and peripheral glial cells) [2] that are associated with several pain conditions. Thus, TRPV4, like other TRP channels, contributes to detecting and transducing pain signals, which makes it a promising target for developing analgesic and anti-inflammatory agents [3]. The TRPV4 channel is modulated with several stimuli such as temperature, hypotonicity, stretch, UV radiation, endogenous lipids, and synthetic agonists and antagonists (see Supplementary Information, Figure S1 for structures of various TRPV4 agonists and antagonists).

**Figure 1:**
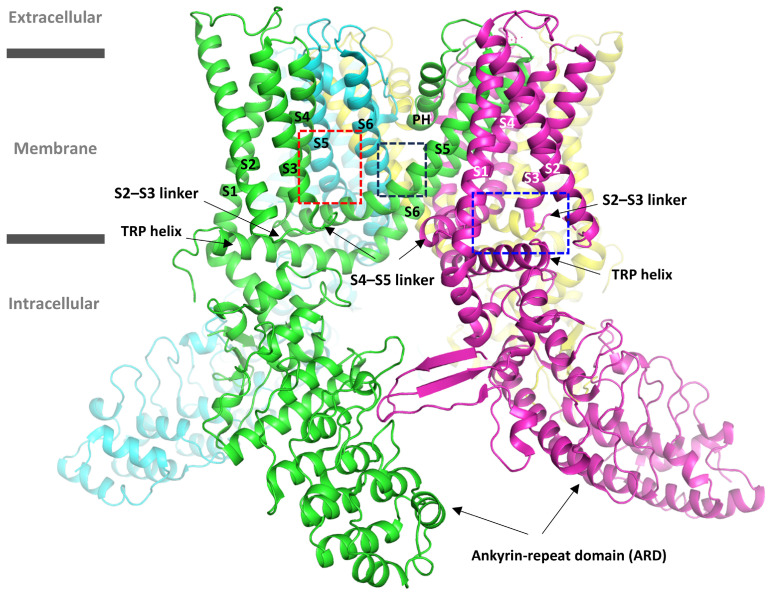
The location of well-known ligand binding sites in TRPV family members is illustrated with the rat TRPV1 (PDB ID: 3J5P) [21] structure (cartoon). The pockets are delineated in blue—voltage-sensing-like domain (VSLD) pocket, red—vanilloid binding site (VBS), and black—cannabidiol (CBD) binding pocket. The domains are labeled, and the four subunits are colored green, cyan, yellow, and magenta.

The general structure of the TRP ion channels is homotetrameric, and each subunit comprises an N-terminal ankyrin-repeat domain (ARD), transmembrane domain (TMD), and a C-terminus (Figure 1). TMD contains six alpha helices (S1–S6), a pore helix (PH), a TRP helix, and several loop linkers. S1 to S4 domains are known as voltage-sensing domains, and S5, PH, and S6 as pore domains. So far, several binding pockets have been identified in the TRPV family (V1–V6). The first site, the vanilloid binding site (VBS), is located between the S3 and S4 helices and the S4–S5 linker of one subunit and S5 and S6 domains of the adjacent subunit (Figure 1) [4]. The VBS site has been identified in TRPV1, TRPV3, and TRPV5 and is shared by both agonists, such as capsaicin [5] and tetrahydrocannabivarin (THCV) [6], as well as antagonists, such as capsazepin [4] and SAF312 [7]. The second site, referred to as the voltage-sensing-like domain (VSLD), is located at the interface of the S1–S4 helical bundle and the TRP helix (Figure 1) and has been proposed as a binding site for the agonist 2-aminoethoxydiphenyl borate (2-APB) in TRPV3 and TRPV6 [8,9] and the antagonist ZINC17988990 [10] in TRPV5. Two additional distinct binding sites for 2-APB have been identified: one at the intracellular side, between the TRP helix and ARD in TRPV3 [6], and the other at the interface, between the S5 helix of one subunit and the S4–S5 linker of the adjacent subunit [11]. Also, another binding site for THCV in TRPV6 has been reported, formed by the S5 and S6 helices of one subunit and the S6 helix of the neighboring subunit (Figure 1) [12]. This site was previously shown to bind the agonist cannabidiol (CBD) in TRPV2 [13], and the same pocket has been reported for the TRPV5 antagonist ZINC9155420 [10]. A hydrophobic pocket between the S3 and S4 helices and the S6 helix of the adjacent subunit was previously reported for the antifungal econazole (an antagonist) in TRPV5 [14]. More recently, another binding site for econazole was identified between the S1, S4, and S5 (adjacent subunit) helices in both TRPV5 [15] and TRPV6 [16].

Among the lipids and other small molecular compounds that modulate TRPV4, the binding sites have been determined; for example, for 5′,6′-epoxyeicosatrienoic acid (5′,6′-EET) [17], phorbol ester 4α-phorbol 12,13-didecanoate (4α-PDD), and GSK1016790A [18] (TRPV4 agonists), as well as for HC-067047 [19] and GSK2798745 [20] (TRPV4 antagonists) (see Supplementary Information, Figure S1 and Table S1). In an attempt to identify the 5′,6′-EET binding site in TRPV4, Berna-Erro et al. [17] generated a human TRPV4 (hTRPV4) model using hTRPV1 in the closed state (PDB ID: 3J5P) [21] as the template. A pocket between the S2–S3 linker (residues K535, F549, and Q550), S4 helix (Y591), and S4–S5 linker (R594) was detected using molecular docking and molecular dynamics (MD) simulations. This pocket was further validated using mutational studies and biological assays. The residues that interact with 5′,6′-EET are highly conserved in other species. The hTRPV4 residues Y591 and R594 are identical in hTRPV1 and V2. On the other hand, the residue corresponding to K535 in TRPV4 is glutamine (Q498) in hTRPV1, which can be the possible reason for hTRPV1 insensitivity to 5′,6′-EET [17].

Doñate-Macian et al. (2022) reported an antagonistic binding pocket for HC-067047, formed by residues from the S2–S3 loop, the S4 helix, and the S5 helix of the adjacent subunit. First, the SiteMap tool was used to identify putative druggable sites in the TRPV4 channel of the Western clawed frog (*Xenopus tropicalis*) (xTRPV4) [19]. Consequent docking studies suggested a favorable binding site that was further explored by MD simulations combined with in silico mutagenesis of the identified key residues. Finally, experimental mutagenesis of the corresponding hTRPV4 residues and inhibition studies confirmed that D546 (S2–S3 loop) and Y591 (S4) have an essential role in the inhibitory activity of HC-067047 at hTRPV4. Residue Y591 has been previously reported to engage in channel gating [22] and interact with 5′,6′-EET [17]. The study by Doñate-Macian et al. (2022) revealed also that D546 is a key residue for the inhibitory effect of RN1734 (a selective TRPV4 antagonist [23], structurally different from HC-067047). Thus, the identified HC-067047 pocket seems to overlap partially with the 5′,6′-EET and RN1734 binding sites.

Botte et al. (2020) [18] predicted the binding mode of 4α-PDD (a weak agonist, EC_50_: 200 nM) using cryogenic electron microscopy (cryo-EM), mutagenesis, and docking studies. Their hTRPV4 cryo-EM structure (PDB ID: 7AA5, resolution 4.18 Å) was the first hTRPV4 structure in a ligand-induced open state and suggested that the 4α-PDD binding site is at the interface between S1, S2, and the TRP helix where the agonist makes a putative hydrogen bond with N474. N474Q (S1 helix), S548V (S2–S3 loop), and R594A (S4–S5 linker) mutations of the residues located in the proposed binding pocket completely abrogated any calcium response to 4α-PDD. The mutagenesis studies revealed that there is an overlap between the 4α-PDD and GSK1016790A (a strong agonist, EC_50_: 2 nM) binding sites within the VSLD.

More recently, many cryo-EM structures of hTRPV4 in different states have been published (see Supplementary Information, Table S1). A cryo-EM structure of a partially closed hTRPV4 in complex with HC-067047 (PDB ID: 8TIF) [24] revealed that like the site for 4α-PDD and GSK1016790A, the HC-067047 binding site is located at the base of the VSLD (S1–S4), surrounded by residues F471 (S1), N474, S477 (S1), Y478 (S1), D546, Y553 (S3), Y591, F592 (S4), and D743 (TRP helix). Consistent with their cryo-EM data and the previous findings, mutations in residues N474, D546, and Y591 decreased the potency of HC-067047 [24]. Another potent and novel TRPV4 antagonist, GSK2798745, also occupied the VSLD site, interacting with aromatic or hydrophobic residues F524 (S2), Y553, Y591, F592, and I744 (TRP helix), as well as polar residues N474, T527 (S2), and D743 [20].

In recent years, some hTRPV4 antagonists and agonists have been developed in the hope of treating, for example, pain, edema, gastrointestinal disorders, and respiratory diseases [3,25]. Inhibiting hTRPV4 may be beneficial, for example, in asthma [25], whereas activating could be useful for the treatment of osteoarthritis [26]. There is also growing evidence that targeting more than one TRP channel simultaneously could be an effective approach for the treatment of certain ailments [27]. For example, both TRPV4 and TRPA1 (another TRP family member that plays a crucial role in various physiological and pathological processes) are involved in painful and/or inflammatory conditions such as colitis, pancreatitis, headache, and chronic cough [28]. Consequently, dual TRPV4/TRPA1 inhibitors could be beneficial in the clinic and, indeed, such compounds have shown promising results in animal models of pain and inflammation [28].

So far, despite all the efforts to find therapeutically effective TRPV4 modulators, only the TRPV4 antagonist GSK2798745 has succeeded in entering clinical studies (Phase I and II) as a drug candidate for several indications [29]. Therefore, in this study, we attempted to discover new potential hTRPV4 antagonists using structure-based and ligand-based drug design approaches. Furthermore, inspired by the multi-channel targeting approach, we explored the putative binding and activity of a small set of natural stilbenoids (a class of polyphenolic compounds) on hTRPV4, some of which have previously demonstrated activity on other TRP channels, including TRPA1 and TRPV1 [30,31].

This work was conducted before the first hTRPV4 structures were published. Thus, in the absence of the experimental hTRPV4 structure, we built an hTRPV4 model based on the closed-state xTRPV4 structure and used it for structure-based virtual screening (VS) by molecular docking of a commercial ion channel compound library. MD simulations were then employed to evaluate the stability of the top-ranked ligand-hTRPV4 complexes. Furthermore, a ligand-based VS of the same library was carried out using a pharmacophore model that was created by structurally aligning two known TRPV4 antagonists. ADMET (absorption, distribution, metabolism, excretion, toxicity) properties of the virtual hits were predicted in silico, and the most promising compounds were tested for their inhibitory activity on hTRPV4 using a fluorescent imaging plate reader (FLIPR^tetra^) calcium assay. In addition to assessing the hTRPV4 effects of the natural stilbenoids with the FLIPR assay, we also studied their putative binding interactions at the ion channel using molecular docking. The results are critically discussed in light of the current structural information on the hTRPV4 structure.

## 2. Results and Discussion

### 2.1. Comparative Modeling of hTRPV4 and Binding Site Prediction

Building the comparative (homology) model of hTRPV4 based on the xTRPV4 cryo-EM structure with 78% sequence identity was rather straightforward, as there were no gaps in the modeling alignment of the modeled regions (residues 148–788 of each subunit) (Supplementary Information, p. S5). Ten models of hTRPV4 were generated with MODELLER, and the model with the best DOPE score (−319,982.78125) and a root-mean-square deviation (RMSD) of 0.649 Å from the Cα atoms of the template was selected for the structure-based virtual screening. The stereochemical quality of the model was comparable with the template with 93.4% of the residues in the most favorable region (87.8% for xTRPV4) and 6.5% in the allowed regions (12.8% in the additional allowed region for xTRPV4) of the Ramachandran plot. Only 0.2% of the model residues were in the disallowed region, one residue from each subunit, and these were found in flexible, unstructured areas, away from the investigated binding sites. After protein preparation (see Methods), only 0.1% of those residues remained in the disallowed area (see Supplementary Information, Figure S2). The model was also stable in a 300 ns MD simulation and did not show significant RMSD fluctuations (Supplementary Information, Figure S3).

To our knowledge, so far, the VSLD site is the only allosteric ligand binding site experimentally observed for TRPV4 (Supplementary Information, Table S1). Neither endogenous nor exogenous ligands have been experimentally observed binding at the VBS of TRPV4. Ruthenium red has, however, been reported to block the TRPV channel pore directly by binding at the extracellular part of the selectivity filter [32]. There is some variation in the residues forming the VSLD cavity among the thermoTRPVs. For example, over 50% of the residues forming the identified 4α-PDD site are weakly or not at all conserved [18]. On the other hand, based on the multiple sequence alignment of representative TRPV family members, the residues of the VBS pocket are highly conserved among these channels (Supplementary Information, Figure S4). The evolutionary conservation of the amino acids in hTRPV4 was also analyzed with ConSurf, which confirmed that the amino acids in the VBS are more conserved compared to those in the VSLD (Supplementary Information, Figure S5A). To identify a putative druggable binding pocket in (the proximity of) the conserved VBS area in our model, we used the SiteMap tool of Schrödinger’s Maestro. Among the predicted sites, a cavity with a SiteScore of 1.271 and a Dscore of 1.400 was selected for the virtual screening. This mostly hydrophobic site is located between the S3, S5, and pore helix domains of one subunit and the S6 helix of the neighboring subunit (Supplementary Information, Figure S6).

In light of the recent structural insights into the TRPV ligand binding sites and the hTRPV4 structure, we retrospectively analyzed our selected binding site. We observed that the pocket we used for virtual screening overlaps (and is bit above) the highly conserved cannabinoid binding site that has been identified for CBD in TRPV2 [13] and for THCV in TRPV6 [12]. The cannabinoid binding site is located just next to the VBS (Figure 1). Another thing revealed from the newly published experimental TRPV4 structures was that the domain arrangement in the TRPV4 open state is similar to the one that has been observed in the other members of the thermoTRPVs, V1–V3 [18,33]. However, although the overall architecture of the thermoTRPVs is similar, the conformation of the VBS and VSLD cavities is not conserved in the closed state of xTRPV4 [33]. Botte et al. [18] reported that although the internal arrangement of the S1–S4 bundle is the same in the closed-state xTRPV4 structure (PDB ID: 6BBJ) and the open-state hTRPV4 structure (PDB ID: 7AA5), the bundle is rotated by approximately 90° in the open state with respect to the closed state. This leads to changes in the interactions between the S1–S4 helices and the nearby domains: the orientations of the TRP helix and the pore domain change relative to the S1–S4 bundle. This rotation of the S1–S4 bundle results in a notable change in the arrangement of domains S3 and S4 from one subunit with respect to the S5 and S6 domains from the neighboring subunit. In the closed state, S3 has moved towards the pore and is in close contact with the S6 domain from the adjacent subunit, while in the open state, the S4 domain is the only one in contact with the S5 and S6 domains. Moreover, it was shown that since the TRP helix in the N terminus rotates about 60° away from the S1 and S2 helices in the closed state, it cannot anymore contribute directly to 4α-PDD binding [18]. Furthermore, the S4–S5 linker shows a random coil structure in the xTRPV4 closed state, while it forms a helix in both open and closed states of all other known TRPV structures, regardless of the species or the subtype [33]. Structural analyses of the many recent hTRPV4 cryo-EM structures in various states have revealed that despite some small-scale conformational changes between the states, the relative positioning of the S1–S4 bundle and the pore domains of hTRPV4 in the open and closed states is similar [24]. This significant conformational change between the open and closed states seems to be unique for xTRPV4 and has so far not been observed in the TRPV4 of other species or other thermoTRPV members (Supplementary Information, Figure S7) [33,34].

Since our comparative model was based on the xTRPV4 structure, this conformational change affects the conformation of the VSLD and VBS sites significantly. The CBD binding site is also somewhat affected by this [35] and so is the cavity identified by SiteMap; the position of S3 differs in hTRPV4 compared to our model, and S5, which is straight in the model, is bent in hTRPV4 (Supplementary Information, Figure S8).

### 2.2. Virtual Screening for the Discovery of Novel hTRPV4 Antagonists

#### 2.2.1. Structure-Based Virtual Screening

To identify novel hTRPV4 antagonists, a structure-based VS campaign by molecular docking was first performed using Glide and GOLD for a focused ion channel compound library of 36,800 molecules. The top hit compounds ranked by the Glide XP GScore (28 compounds) and GOLD fitness score (50 compounds) were subjected to binding free energy calculations using the molecular mechanics–generalized Born surface area (MM-GBSA) approach. Ten hits (Table 1, Figures S9 and S10) with the highest binding free energy and unique structures were selected for the MD simulations to evaluate the binding stability of the predicted binding complexes.

**Figure 2:**
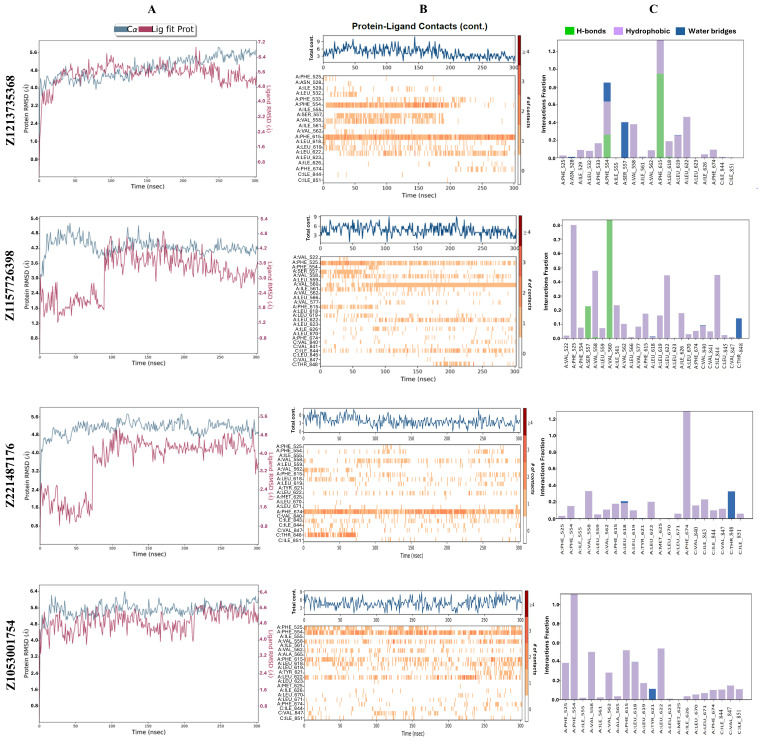
The molecular dynamics (MD) simulation analysis of compounds Z1213, Z1157, Z2214, and Z1053. (**A**) RMSD plot for the hTRPV4 model in complex with the ligand. (**B**) The number of total ligand–protein contacts (top panel) and the residues that interact with the ligand in each trajectory frame (persistency of the interaction) during the 300 ns MD simulation (lower panel). (**C**) The interaction fraction between the specific residues and the ligand (1.0 = interaction is maintained 100% of the simulation time; >1.0 = residue can form multiple interactions with the ligand).

**Figure 3:**
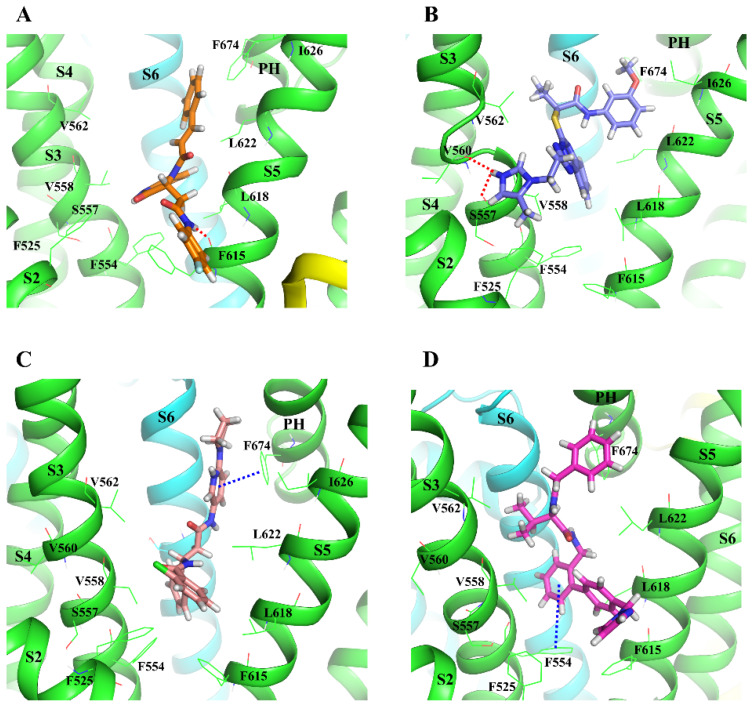
The binding pose of Z1213 in orange stick (**A**), Z1157 in purple stick (**B**), Z2214 in salmon stick (**C**), and Z1053 in magenta stick (**D**) at the predicted binding site of the hTRPV4 model at the end of a 300 ns MD simulation. The hTRPV4 model is represented in a cartoon (subunits colored differently). Atom color code: nitrogen—blue; oxygen—red; fluorine—light blue; hydrogen—white. The key residues (in lines) and the TM domains are labeled. Interaction color code (dashed lines): H-bond—red; π-π—blue.

During the 300 ns MD simulation compound, Z30995330 (Z3099) moved away from the binding pocket toward the extracellular part of the channel, while all the other compounds stayed in the pocket. The RMSD of the protein Cα-atoms for these ligand–protein complexes reached an equilibrium during the 300 ns simulation, except for the complexes with compounds Z1213735368 (Z1213) (Figure 2A) and Z229153032 (Z2291). While there were no significantly high ligand RMSD values at the end of the successful simulation trajectories, suggesting a reasonably stable pose, the rotatable rings at the two ends of some of the compounds were not stably bound but rotated around. The protein–ligand interaction analysis revealed that all the hits, except compounds Z3099, Z169988240 (Z1699), and Z51867077 (Z5186), could form long-term interactions (an interaction that occurs over 80% of the simulation time) with the protein. Compounds Z1053001754 (Z1053) (Figure 2B,C and Figure 3D) and Z31055919 (Z3105) made hydrophobic interactions with F554 in the S3 helix, while compounds Z2291, Z29940381 (Z2994), and Z221487176 (Z2214) (Figure 2B,C and Figure 3C) interacted with F674 in the PH. Only hits Z1213 and Z1157726398 (Z1157) could make a hydrogen bond with F615 (S5 helix) and V560 (S3 helix), respectively (Figure 2B,C and Figure 3A,B). The ligand Z1157 had also an extra hydrophobic interaction with F525 in the S2 helix (Figure 2B,C). Both residues F554 and F674 are conserved in the TRPV family, while residues F525, V560, and F615 were not according to the multiple sequence alignment (Supplementary Information, Figure S4). The persistent hydrophobic interaction of compounds Z1053 and Z2214 (which both had an interaction fraction over one, indicating several contacts with the residue) was visually inspected, and we observed that the interaction for compound Z2214 (Figure 2C) is steadier and firmer (without breaks during the simulation) than compound Z1053 (Figure 2B). Compounds Z1213, Z1157, Z2214, and Z1053 that had the longest-lasting interactions with the hTRPV4 model (either hydrophobic or hydrogen bond interactions) (Figure 2B,C) were selected for the next screening step to evaluate their ADMET properties.

#### 2.2.2. Ligand-Based Virtual Screening

**Figure 4:**
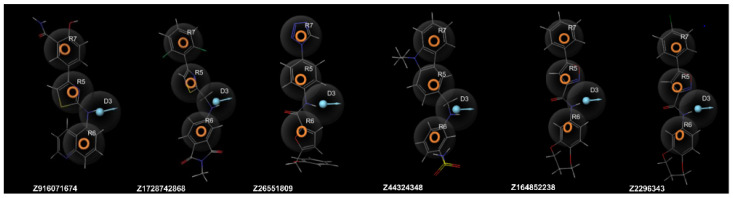
The alignment of the ligand-based virtual screening hits with the pharmacophore model that was used for the screening. Pharmacophore features: orange rings—aromatic rings (R); a blue ball with an arrow—hydrogen bond donor (D).

In addition to the structure-based approach, we generated a ligand-based pharmacophore using two known TRPV4 antagonist structures (GSK205 and HC-067047) and used that to screen through the Enamine library to find potential TRPV4 antagonists. A pharmacophore with four features, three aromatic rings, and one hydrogen bond donor, with a PhaseHypoScore value of 0.766718, was chosen for the ligand-based VS (Supplementary Information, Figure S11). Altogether, six hit compounds with a Phase fitness score over two were selected for the ADMET prediction (Figure 4).

#### 2.2.3. Prediction of ADMET Properties

An ADMET prediction was performed for ten chosen hits from the two VS screening approaches altogether (four hits from the structure-based VS and six from the ligand-based VS) (Table 2). All the compounds belonged to the toxicity class 4 or 5 (classes 1–6, 1—fatal; 6—non-toxic) and showed moderate solubility and good skin permeation. All the hits were predicted to have a high absorption from the gastrointestinal (GI) tract, except compound Z916071674 (Z9160). Compounds Z1213, Z2214, Z164852238 (Z1648), and Z229634356 (Z2296) were predicted to permeate the blood–brain barrier (BBB). Hits Z1728742868 (Z1728) and Z2214 were not predicted to be hepatotoxic, immunotoxic, mutagenic, carcinogenic, or cytotoxic. Compounds Z443243482 (Z4432) and Z1053 might be carcinogenic, and Z1213 may be immunotoxic. From the ligand-based VS hits, compound Z4432 with the highest LD_50_ value (5000 mg/kg), minimum toxicity, and no BBB permeability, and compound Z1728 with no toxicity, carcinogenicity, or mutagenicity, were chosen for the biological assay. The results from the ADMET prediction were fairly similar for all four hits from the structure-based VS. Therefore, compounds Z1213, Z1157, and Z2214 were selected for the biological study according to the longest-lasting hydrogen and hydrophobic bonds they formed with the hTRPV4 model during the MD simulations.

### 2.3. Biological Activity of the Virtual Screening Hits

**Figure 5:**
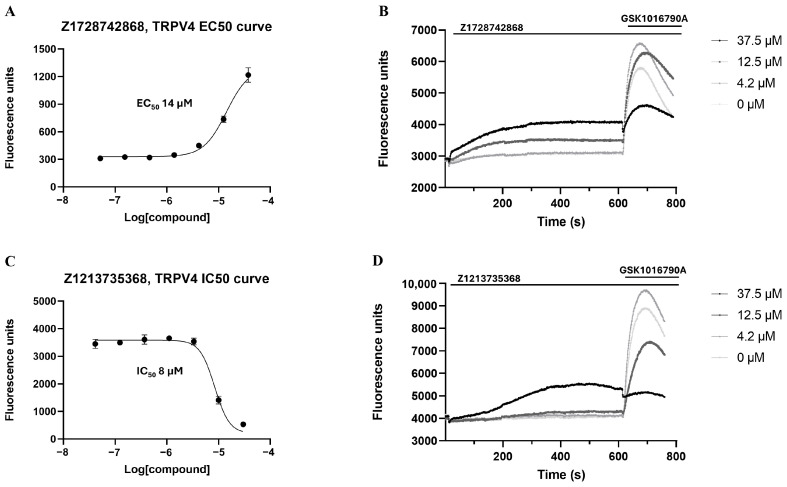
(**A**) The concentration–response curve for compound Z1728. (**B**) Compound Z1728 showed a concentration-dependent increase in [Ca^2+^] with relatively slow kinetics and effectively inhibited the response induced by the TRPV4 agonist (500 nM GSK1016790A) at the highest test concentration. (**C**) The concentration–response curve for compound Z1213. (**D**) At a high concentration, compound Z1213 gradually raised [Ca^2+^] levels in the FLIPRtetra experiment. The compound’s inhibitory effect on the TRPV4 agonist (500 nM GSK1016790A) was observed in a concentration-dependent manner.

Hits Z4432, Z1728, Z1213, Z1157, and Z2214 were selected to examine their effect on hTRPV4 using the FLIPR assay. This study showed that compound Z1728 could evoke an increase in intracellular calcium with the EC_50_ of 14 µM in a concentration-dependent manner (Figure 5A), while the others, except Z2214, showed this response only at a high concentration (37.5 µM). Compound Z1728 could slowly increase the fluorescence signal. However, this increase was not as fast and robust as that with GSK1016790A (a hTRPV4 agonist). Compound Z1728 also inhibited the effect of GSK1016790A at a concentration of 37.5 µM (Figure 5B). All the hits showed an inhibitory effect on hTRPV4 at 30 µM (Supplementary Information, Figure S12). However, only compound Z1213 (IC_50_: 8 µM) (Figure 5C) inhibited the response evoked by GSK1016790A at a concentration of 10 µM without any agonistic activity on the channel. At a high concentration (30 µM), hit Z1213 evoked a slow increase in the fluorescence signal. However, it could also reverse the effect of GSK1016790A (Figure 5D).

Based on the biological data and the predictive in silico study, Z1213 and Z1157, which formed stable hydrogen bonds with the channel model during the MD simulations, can inhibit hTRPV4 more effectively than Z2214 (IC_50_ > 30 µM), which was predicted to have only some short-lived water-mediated hydrogen bonds and a long-lasting hydrophobic interaction.

### 2.4. Assessing the Putative Binding Site and Biological Activity of the Natural Stilbenoids

Whilst working on this article, the first cryo-EM structure of hTRPV4 (PDB ID: 7AA5) was released by Botte et al., and the density (but not clear coordinates) of 4α-PDD was reported in the VSLD binding pocket [18]. The authors further predicted the 4α-PDD binding site by mutational and docking studies. We then used this information when we explored the putative binding interactions of a set of natural stilbenoids at hTRPV4. Thus, pinosylvin, pinosylvin monomethyl ether (PsMME), resveratrol, isorhapontin, and astringin were docked into two specified binding pockets (our SiteMap-identified pocket and the 4α-PDD site) in the hTRPV4 model. Both XP GScore and the Prime/MM-GBSA values indicated poor binding for pinosylvin, PsMME, and resveratrol at both binding sites (Supplementary Information, Table S2). In contrast, astringin and isorhapontin, being larger molecules, exhibited better docking scores and binding free energies at the 4α-PDD binding site due to their increased interaction potential compared to the aglycone compounds (Supplementary Information, Table S2). Astringin and isorhapontin adopted a mutually similar pose and formed hydrogen bonds with the backbone of N541 (S2–S3 linker) (Supplementary Information, Figure S13), similar to the reference compound 4α-PDD in the docking study by Botte and co-workers [18], who used the cryo-EM structure of hTRPV4. However, these glucosides did not engage in any other interactions predicted for 4α-PDD by the mutational and modeling studies by Botte et al. [18]. On the other hand, when the reference compound was docked to our hTRPV4 model, the resulting pose resembled closely the pose obtained by Botte and co-workers. Moreover, in the pose obtained by us, 4α-PDD formed interactions with some of the key residues identified in Botte’s study (N474 and Q550).

Furthermore, we studied the pharmacological effects of the natural stilbenoids on hTRPV4. Although some activity was detected at high concentrations, the signals appeared relatively weak compared to the TRPV4 agonist control (GSK1016790A). None of the compounds exhibited a significant inhibitory effect on hTRPV4 (Supplementary Information, Figure S14). This is in line with the in silico study, as pinosylvin, PsMME, and resveratrol were predicted to bind poorly to hTRPV4. While the stilbenoid glucosides (astringin and isorhapontin) were predicted to bind better in the hTRPV4 model, they also remained inactive in vitro. The inactivity of astringin and isorhapontin could be attributed to their hydrophilic glucoside structure, which may hinder their ability to pass through the cell membrane and reach the binding site. Alternatively, their aglycone structures do not bind to the channel [36].

### 2.5. Molecular Docking of the Hit Compounds to the 4α-PDD Binding Site at the VSLD

We continued using our hTRPV4 model when we further explored the possibility of an alternative binding site for our structure-based VS hit compounds and a putative binding site for the ligand-based VS hits. The five biologically tested hTRPV4 hits, GSK205, HC-067047, and the reference compound (4α-PDD) were docked into the 4α-PDD pocket at the VSLD in the hTRPV4 model using GOLD and Glide. The free energy of binding was also calculated to be able to compare the docking results from the different tools. The hits from the structure-based VS could form a hydrogen bond with N474 (S1 helix)-like 4α-PDD and their Glide XP/GOLD fitness scores and binding free energies were comparable with the reference ligand (Supplementary Information, Table S3). On the other hand, the hits from the ligand-based VS and the pharmacophore template molecules GSK205 and HC-067047 showed less strong binding to this binding pocket compared to the SiteMap-predicted pocket (Supplementary Information, Table S3). Moreover, the stability of the binding interactions of compound Z1213 (as the most potent hit) in the 4α-PDD binding pocket was analyzed by three parallel 400 ns MD simulations, and its free energy of binding was calculated before and after the simulations. Although the ligand stayed in the pocket during all the simulations, only in one out of three simulations did its hydrogen bond with N474 last more than 80% of the time (Supplementary Information, Figure S15).

### 2.6. Post-Study Analysis in Light of More Recent Structural Data

Due to the later observed differences between the open-state hTRPV4 and our model, which was based on the closed-state xTRPV4 structure, we also examined the binding interactions of the three structure-based VS hits and the stilbenoids in the 4α-PDD binding pocket of the experimental hTRPV4 structure (PDB ID: 7AA5) (Supplementary Information, p. S22). Although the hits formed a hydrogen bond with N474, their predicted binding free energies were lower than those observed in our model (Supplementary Information, Table S4). While the hit compounds occupied the same site as our docked 4α-PDD, they could not form interactions with all the other key residues predicted to interact with 4α-PDD (T527, K530 (S2), D531 (S2), S747 (TRP helix), and D743). Furthermore, although the stilbenoids formed several hydrogen bonds and hydrophobic interactions with the target, including similar interactions (N474, T527, Y591, F592 and R594) to the other VSLD-binding TRPV4 modulators (4α-PDD [18], GSK2798745 [20], HC-067047 [24]), they exhibited poor binding free energy at this site (Supplementary Information, Table S4).

## 3. Materials and Methods

### 3.1. Comparative Modeling of hTRPV4

The amino acid sequences of both human (UniProtKB: Q9HBA0) and Western clawed frog (UniProtKB: F7BWY7) TRPV4 were obtained from the UniProt Knowledgebase (https://www.uniprot.org). The three-dimensional (3D) structure of the template (xTRPV4, PDB ID: 6BBJ, resolution 3.8 Å [34]) was retrieved from the Protein Data Bank. The modeling alignment was generated with Clustal Omega [37] (Supplementary Information, p. S4) and manually formatted for the MODELLER (v.10) [38] program that was used for creating the comparative model of hTRPV4. The template contained coordinates only for the xTRPV4 residues 144–784 and lacked the structural data of three flexible loops (res. 531–535, 636–656, 763–765) in each subunit. We thus modeled the corresponding hTRPV4 residues 148–788, including the missing loops [39], using MODELLER’s standard automated comparative modeling procedure for all four subunits. Out of the ten generated alternative models, the one with the most negative Discrete Optimized Protein Energy (DOPE) score [40] was chosen for further studies. The stereochemical properties of the model were evaluated with the Ramachandran plot using PROCHECK [41], and the model was visually inspected using the PyMOL Molecular Graphics System (version 2.5.5 Schrödinger, LLC, New York, NY, USA). A 300 ns MD simulation was performed with Desmond [42] as implemented in the Maestro Molecular Modeling Suite (Schrödinger, LLC, New York, NY, USA, 2020) to examine the stability of the model (see Section 3.7 below).

### 3.2. Active Site Prediction

The SiteMap tool in Maestro was used to locate putative binding cavities in the hTRPV4 model. The default settings in SiteMap were used; at least 15 site points were required per reported site. The putative binding sites were evaluated visually, and the pocket for virtual screening was selected based on the favorable values of the two druggability assessment scores, SiteScore and Dscore [43]. In addition, the existing literature and structural data of the known ligand binding sites in the TRPV family were used to guide the pocket selection. The evolutionary conservation of the hTRPV4 residues was analyzed using the ConSurf server [44,45], as well as through a multiple sequence alignment of the hTRPV4 sequence with the representative TRPV family members that had available structural data at that time: UniProtKB IDs: O35433 (rat TRPV1), G1SNM3 (rabbit TRPV2), Q8NET8 (hTRPV3), Q9XSM3 (rabbit TRPV5), and Q9H1D0 (hTRPV6).

### 3.3. Preparing Protein and Ligands

The human TRPV4 model was prepared for docking with the Protein Preparation Wizard in Maestro [46] using the OPLS3e [47] force field. Missing hydrogen atoms were added, the H-bond network was optimized, and a restrained minimization was first executed for the hydrogen atoms only and then for the heavy atoms until they reached an average of a 0.3 Å root-mean-square deviation (RMSD). A commercially available database of 36,800 molecules (Enamine Ion Channel molecules) was downloaded from Enamine’s website (https://enamine.net/compound-libraries/targeted-libraries/ion-channel-library, accessed on 24 May 2020). The structures of natural stilbenoids (pinosylvin, pinosylvin monomethyl ether, resveratrol, isorhapontin, astringin) and the reference compounds (4α-PDD, GSK205, and HC-067047) were retrieved from the ChemSpider database (www.chemspider.com). All compounds were prepared using the LigPrep module of Maestro (Schrödinger Releases 2020-2, 2020-4, 2022-1; LigPrep, Schrödinger, LLC, New York, NY, USA). Possible protonation states of the compounds were created using the OPLS3 [48] (stilbenoids) or the OPLS3e force field at pH 7 ± 2. At most, 32 stereoisomers were produced per ligand in the Enamine database.

### 3.4. Structure-Based Virtual Screening by Molecular Docking

The selected docking site was defined with the Receptor Grid Generation tool of Maestro. The enclosing cubic grid was centered at coordinates (X:154.79, Y:147.11, Z:166.45) guided by the SiteMap site points, and the length of the ligands to be docked was limited to 18 Å. The receptor thiol or hydroxyl groups in the binding site were allowed to rotate.

VS by molecular docking was carried out with the Glide tool (Glide, Schrödinger, LLC, New York, NY, USA, 2020) [49], employing the Virtual Screening Workflow of Maestro. The Glide high-throughput virtual screening (HTVS) mode was used for the initial docking of the database. At most, five poses per ligand were generated, and the top 10% of the compounds based on the docking score were selected for the next screening step, which was the redocking using the standard precision (SP) algorithm. Finally, 10% of the highest-ranked molecules from the previous step were redocked using the extra-precision (XP) mode, and the hits with a Glide XP GScore lower than −9 kcal/mol were chosen for the binding free energy calculations with the Prime/MM-GBSA module of Maestro [50].

Furthermore, two rounds of VS by molecular docking of the Enamine database were conducted using the GOLD (Genetic Optimisation for Ligand Docking) program (Cambridge Crystallographic Data Centre, version 2021.2.0) [51]. The docking site was centered to the precise coordinates previously employed in Glide, and the size of the grid box was set to 18 Å × 18 Å × 18 Å. ChemPLP (ChemScore [52,53] + Piecewise Linear Potential [54]) was chosen as the fitness function. The automatic, ligand-dependent genetic algorithm (GA) parameter settings were used. In the first round of screening, the GA search efficiency was set at 10%, and at most, five poses were generated per ligand. The low search efficiency is recommended for library screening and corresponds to the HTVS mode of Glide as the speed of docking is increased at the cost of the sampling of the search space (i.e., prediction accuracy). In this round, 500 best-ranking molecules were chosen, and their binding mode was inspected visually. Since the defined docking grid also covered other potential binding sites than the targeted one, we then selected only the molecules that were docked into the targeted cavity for the next round of screening. The selected molecules were redocked using a search efficiency of 100%, which allows the GA to run an optimal number of genetic operations to explore the search space of a ligand [55]. Finally, the 50 best-ranking molecules according to the GOLD fitness score were chosen for the binding free energy calculations using the molecular mechanics–generalized Born surface area (MM-GBSA) approach (see below).

### 3.5. Molecular Docking of Natural Stilbenoids

Molecular docking of the set of natural stilbenoids (pinosylvin, pinosylvin monomethyl ether, resveratrol, isorhapontin, astringin) was conducted using Glide in XP mode. The putative binding interactions of these compounds at the hTRPV4 model were evaluated in two binding sites: the virtual screening site predicted by SiteMap (near the VBS and the CBD site) and the recently reported agonistic pocket for 4α-PDD at the VSLD [18]. The docking site at the VSLD of the hTRPV4 model was defined using the Receptor Grid Generation tool, centered on residues N474, Y591, R594, Y553, Y556, and S747, which were selected based on mutational and modeling studies [18]. The ligand diameter midpoint box was set to 10 Å × 10 Å × 10 Å, and the length of ligands to be docked was limited to 15 Å. Up to five poses per ligand were generated, and the best pose for each ligand was selected for calculating the binding free energy using the MM-GBSA method.

### 3.6. MM-GBSA Binding Free Energy Calculation

The free energy of binding for the top-ranked compounds from both Glide and GOLD screening and the best poses of the natural stilbenoids was calculated with the Prime/MM-GBSA module of Maestro (Schrödinger Releases 2020-2, 2022-1, 2023-2; Prime, Schrödinger, LLC, New York, NY, USA, 2020) using the VSGB solvation model [56] and the OPLS3e (database hits) or the OPLS4 [57] (stilbenoids) force field, keeping all binding site residues fixed. Flexible sampling was performed by minimization of the complex.

### 3.7. MD Simulation Analysis

The top ten hTRPV4 ligand docking complexes based on the calculated binding free energy (Prime/MM-GBSA ΔG-bind value) from the structure-based VS were chosen for an MD simulation analysis by the Desmond MD engine [42] implemented in Maestro (Schrödinger Release 2020-2: Desmond Molecular Dynamics System, D. E. Shaw Research, New York, NY, USA, 2020. Maestro-Desmond Interoperability Tools, Schrödinger, New York, NY, USA, 2020). The simulation systems consisting of the protein–ligand complexes embedded in a lipid membrane (POPC, 1-palmitoyl-2-oleoyl-sn-glycero-3-phosphocholine) with an explicit solvent (TIP3P water) [58] and a physiological salt concentration (0.15 M) were generated with the System Builder tool of the Desmond module (Supplementary Information, Figure S16). An orthorhombic box with a 10 Å buffer distance between the solute and the simulation box edge was used, and the systems were neutralized by adding Cl^−^ counter ions. Periodic boundary conditions and the OPLS3e force field were applied. A cut-off radius of 9 Å was used for short-range interactions, and the long-range electrostatics were handled with the Partial Mesh Ewald [59] or the U-series method [60]. After the system relaxation, the production simulations were run for 300 ns at a constant temperature (300 K) and pressure (1.01325 bar) using the default temperature and pressure coupling methods as implemented in the Desmond module of Maestro.

An MD simulation system with the hTRPV4 model in a complex with compound Z1213 at the VSLD pocket embedded in a POPC membrane and solvated with TIP3P water was set up. The system was neutralized by adding Cl^−^ counter ions, and after the system relaxation, three parallel production simulations by Desmond were run for 400 ns at a constant temperature (300 K) and pressure (1.01325 bar) using randomized velocities according to our previously reported simulation protocol [61].

### 3.8. Ligand-Based Virtual Screening Using a Pharmacophore Model

A pharmacophore model was created based on the structures of two known TRPV4 antagonists: GSK205 and HC-067047. The Phase tool [62] of Maestro (Phase, Schrödinger, LLC, New York, NY, USA, 2020) was employed to develop a pharmacophore hypothesis (a model), and the model was used to screen the Enamine database for matching compounds. First, the best alignment and common features of the molecules were identified. PhaseHypoScore was used to rank the resulting hypotheses that fulfilled the given criteria (Supplementary Information, Figure S11), and the best-ranked hypothesis was selected for the screening. The ligand database was converted to the Phase-compatible format [62] in Maestro before the screening. The number of hits from the screening was limited to 150, and the output ligand conformers were minimized. The ligands were ranked by the Phase fitness score (range: −1.0–3.0 from the worst to the best; it is a linear combination of the site and vector alignment scores, as well as the volume score) based on how well they aligned with the features of the pharmacophore hypothesis. The top-ranked ligands were submitted to the ADMET properties prediction.

### 3.9. Prediction of Pharmacokinetic and Toxicological Properties

The ADMET properties of the hits from both the structure- and ligand-based VS were predicted using the SwissADME [63] and ProTox II [64] tools. SwissADME was used to predict solubility, synthetic accessibility, gastrointestinal (GI) absorption, permeability through the blood–brain barrier (BBB) and skin, inhibitory effect on drug-metabolizing cytochrome P450 (CYP) enzymes, and if a compound is a P-glycoprotein (P-gp) substrate or not. The lethal dose (LD_50_) value, toxicity class (class 1: fatal to class 6: non-toxic), hepatotoxicity, immunotoxicity, mutagenicity, cytotoxicity, and carcinogenicity were predicted with ProTox II.

### 3.10. Biological Assay

The TRPV4 pharmacology of the hit compounds (Enamine) and the natural stilbenoids (purity > 95%, extracted at the Laboratory of Organic Chemistry at Åbo Akademi University) [65,66] was studied with the fluorometric imaging plate reader FLIPR^tetra^ (Molecular Devices, San Jose, CA, USA) using a dual addition protocol, in which a compound’s agonism is initially measured and then, subsequently, a reference agonist is added to assess the compound’s possible antagonism. The studies were conducted in a 96-well plate format using HEK-293 cells (ATCC) that were transiently transfected with the human TRPV4 plasmid (Origene, Rockville, MD, USA, cat. SC309684). HEK cells were used as they are easy to transfect, and TRPV4 overexpressing cells provide a robust system to study TRPV4-mediated calcium responses with a wide signal window. In the experiments, the cells were loaded with a Calcium 4 Assay reagent (Molecular Devices) to enable intracellular calcium measurements. The study protocol was initiated by dispensing a concentration series (0.05–37.5 µM) of the test compounds on cells by FLIPR^tetra^, and the agonism was recorded for 10 min followed by the second addition, 500 nM of the known TRPV4 agonist GSK1016790A (Sigma-Aldrich, St. Louis, MI, USA, cat. G0798). The antagonism of the test compounds was measured for 2 min. All the experiments were performed at 37 °C. The excitation and emission were measured at wavelengths of 470–495 nm and 515–575 nm, respectively.

In compound agonism and antagonism analysis, the fluorescence values were normalized by subtracting the baseline value from the maximum value of the first 10 min and of the last 2 min measured for each well, respectively. EC_50_ and IC_50_ values were determined from dose–response curves using a nonlinear regression curve fit by Prism (version 9.1.0). Dose–response curves were constructed from a mean of 3 separate wells at each compound concentration.

## 4. Conclusions

In this study, we have used both structure- and ligand-based VS approaches to search for potential TRPV4 antagonists. First, in the absence of a hTRPV4 structure, we created a comparative model of hTRPV4 and screened a library of 36,800 small molecules by docking them into a SiteMap-predicted binding site near the VBS. Ten promising hits with the best XP GScore/GOLD fitness score values, good binding free energies, and unique structures were chosen for the MD simulation analysis. Three of the hit compounds showed favorable interactions with the hTRPV4 model during the simulation, and their pharmacological activity on hTRPV4 was evaluated by the FLIPR^tetra^ assay. Second, a ligand-based VS was conducted based on a pharmacophore model created using GSK205 and HC-067047, assuming that these two known antagonists occupy the same (unknown) binding pocket. Finally, the pharmacological activity of two hits from the pharmacophore-based screening with high fitness score values and favorable predicted ADMET properties were tested on hTRPV4.

All hits could increase the intercellular Ca^2+^ concentration at high concentrations, except compound Z2214. Although the hit Z1728 activated the channel with an EC_50_ of 14 µM, this activation was not as fast and strong as with a potent agonist (GSK1016790A). On the other hand, all hits could reverse the effect of GSK1016790A at a high concentration with the IC_50_ from 8 µM for ligand Z1213 to over 30 µM for Z2214. The hit Z1213 showed better potency than the others and inhibited the response induced by GSK1016790A at a concentration of 10 µM. Of note, intracellular calcium concentration can be increased either directly or indirectly through the activation of the TRPV4 ion channel.

Interestingly, despite the different conformations of the used hTRPV4 model compared to the recent experimentally revealed hTRPV4 structures, two out of the three tested structure-based VS hits showed inhibitory activity at hTRPV4. One possible reason for the positive outcome may be the focused ion channel compound library that we screened. The novel hTRPV4 antagonist, compound Z1213, was identified through the structure-based VS, and it was predicted to have favorable binding to the SiteMap-identified site. If its actual binding site would not be this predicted site, the recently experimentally identified 4α-PDD site at the VSLD could be an alternative. In addition to agonists, such as 4α-PDD, antagonists have also recently been shown to bind at the VSLD (Table S1). However, the retrospective docking and simulation studies of compound Z1213 at the 4α-PDD pocket in our hTRPV4 model, or docking it to the same site in the experimental structure, did not clarify the binding pocket for the antagonist. Other putative binding sites could be the VBS or the CBD site, which are also shared between the agonists and antagonists in the TRPV family. However, no structural data on ligands binding to the VBS or the CBD pocket at hTRPV4 are available to date. Further testing of the putative binding site of Z1213 requires combining in silico analysis with binding assays, mutational studies, or preferably, structural biology to determine the ligand–protein complex.

Finally, for the first time, the activity of natural stilbenoids on hTRPV4 was examined through both in silico and in vitro studies. Given that some of the stilbenoids have shown activity on TRPA1 [67], we tested the hypothesis that they could act as dual TRPA1/TRPV4 modulators. However, the stilbenoids were inactive on TRPV4, which was also consistent with our in silico findings (with the assumption that the binding site might be either the predicted site near the VBS or the VSLD pocket). Overall, the results of this study give insight into the structural determinants of hTRPV4 modulation and may facilitate further efforts in developing therapeutic hTRPV4 ligands.

## Figures and Tables

**Table 1:** The ten hit compounds selected from the structure-based virtual screening either with their Glide or GOLD docking score and calculated binding free energies.

Enamine ID	Docking Score ^a^ (Glide ^b^/GOLD ^c^)	Prime/MM-GBSA ΔG-Bind (kcal/mol)
Z1053001754	−9.889 ^b^	−65.87
Z169988240	−9.783 ^b^	−63.77
Z31055919	−9.160 ^b^	−54.26
Z1213735368	−9.491 ^b^	−56.91
Z30995330	82.083 ^c^	−61.80
Z229153032	76.829 ^c^	−63.45
Z1157726398	77.728 ^c^	−61.29
Z29940381	83.858 ^c^	−70.22
Z51867077	82.111 ^c^	−67.91
Z221487176	82.300 ^c^	−60.48

^a^ The top docking scores according to each docking software were used to select the initial hits; the compounds with the best MM-GBSA energies were then selected from both the initial hit lists: four from Glide docking (from the twenty-eight best-ranked compounds) and six from GOLD docking (from the fifty best-ranked compounds); ^b^ Glide XP GScore (kcal/mol)—the more negative the value, the better; ^c^ GOLD fitness score—the more positive the value, the better.

**Table 2:** ADMET properties of the hits from structure-based and ligand-based virtual screening.

Compound Properties	Compound ID									
	Z1053	Z1213	Z1157	Z2214	Z9160	Z1728	Z2655	Z4432	Z1648	Z2296
Phase fitness score ^a^	-	-	-	-	2.149	2.056	2.052	2.038	2.028	2.024
LD_50_ ^b^ (mg/kg)	2290	2500	500	1800	1000	350	2000	5000	4000	500
Toxicity class	5	5	4	4	4	4	4	5	5	4
Hepatotoxicity	Inactive	Inactive	Inactive	Inactive	Active	Inactive	Active	Inactive	Active	Active
Carcinogenicity	Active	Inactive	Active	Inactive	Active	Inactive	Active	Active	Active	Inactive
Immunotoxicity	Inactive	Active	Active	Inactive	Active	Inactive	Inactive	Inactive	Inactive	Inactive
Mutagenicity	Inactive	Inactive	Active	Inactive	Inactive	Inactive	Inactive	Inactive	Active	Active
Cytotoxicity	Inactive	Inactive	Inactive	Inactive	Inactive	Inactive	Inactive	Inactive	Inactive	Inactive
Solubility ^c^ (logS) ESOL ^d^/QSPR ^e^	−5.57/ −6.20	−4.53/ −4.61	−5.62/ −6.86	−5.00/ −5.33	−4.83/ −6.24	−4.52/ −4.89	−4.09/ −4.62	−4.70/ −5.17	−4.22/ −4.49	−4.82/ −5.14
GI ^f^ absorption	High	High	High	High	Low	High	High	High	High	High
BBB ^g^ permeation	No	Yes	No	Yes	No	No	No	No	Yes	Yes
Skin permeation Log Kp (cm/s)	−5.77	−6.58	−6.17	−6.18	−5.79	−6.30	−6.40	−6.00	−6.03	−5.79
P-gp ^h^ substrate	Yes	No	Yes	Yes	No	No	No	No	Yes	No
CYP ^i^ 1A2 inhibitor	No	No	No	Yes	Yes	Yes	Yes	Yes	Yes	Yes
CYP2C19 inhibitor	Yes	Yes	Yes	Yes	Yes	Yes	Yes	Yes	Yes	Yes
CYP2C9 inhibitor	Yes	Yes	Yes	Yes	Yes	Yes	Yes	Yes	Yes	Yes
CYP2D6 inhibitor	Yes	Yes	Yes	Yes	Yes	No	No	Yes	Yes	Yes
CYP3A4 inhibitor	Yes	Yes	Yes	Yes	Yes	Yes	Yes	Yes	Yes	Yes
Synthetic accessibility ^j^	3.78	3.72	4.41	3.53	2.94	2.94	3.05	2.73	3.16	3.15

^a^ score range: −1.0–3.0; ^b^ median lethal dose; ^c^ insoluble < −10 < poorly soluble < −6 < moderately soluble < −4 < soluble < −2 < very soluble < 0 < highly soluble; ^d^ estimated solubility; ^e^ quantitative structure–property relationships; ^f^ gastrointestinal; ^g^ blood–brain barrier; ^h^ P-glycoprotein; ^i^ cytochrome P450; ^j^ accessibility range: 1–10 (easy to difficult).

## Data Availability

Data are contained within the article/Supplementary Materials; further inquiries can be directed to the corresponding authors.

## Supplementary Materials

The following supporting information can be downloaded at https://www.mdpi.com/article/10.3390/molecules30010100/s1, Figure S1: Structures of TRPV4 agonists and antagonists; Table S1: The cryo-EM structures of TRPV4 in the Protein Data Bank; p. S5: Clustal O (1.2.4) multiple sequence alignment; Figure S2: The Ramachandran plots; Figure S3: RMSD evolution of the hTRPV4 model during a 300 ns MD simulation; Figure S4: Multiple sequence alignment; Figure S5: Identifying a ligand binding site in the hTRPV4 model; Figure S6: The SiteMap result for the pocket that was selected for structure-based virtual screening; Figure S7: Superposition of the TM domains of the hTRPV4 model and the experimental structure of hTRPV4 (PDB ID: 7AA5); Figure S8: The conformation of the SiteMap-predicted binding site; Figure S9: Chemical structures of the top-ranked compounds from the structure-based virtual screening by molecular docking; Figure S10: The docked poses of the selected virtual hit compounds in the predicted binding site of the hTRPV4 model; Figure S11: The pharmacophore used for virtual screening of TRPV4 inhibitors; Figure S12: IC_50_ curve for the virtual screening hits at hTRPV4; Table S2: The molecular docking results of the natural stilbenoids in the hTRPV4 model; Figure S13: The docking poses of the natural stilbenoids and the known reference compounds; Figure S14: EC_50_ and IC_50_ curves for the stilbenoids at hTRPV4; Table S3: Predicted binding free energies of the biologically active hTRPV4 hits; Figure S15: The MD simulation analysis of Z1213 in the 4α-PDD binding site of the hTRPV4 model; p. S22: Molecular docking study using the cryo-EM structure of hTRPV4; Table S4: Predicted binding free energies of the structure-based virtual screening hits and the natural stilbenoids at the 4αPDD binding site in the hTRPV4 model and the experimental hTRPV4 structure (PDB ID:7AA5); Figure S16: The MD simulation system [68,69,70,71].
