# Supplementary material for: Structure- and Ligand-Based Virtual Screening for Identification of Novel TRPV4 Antagonists
Source: Molecules. 2024 Dec 30;30(1):100. doi: 10.3390/molecules30010100 (PMC11722135; doi:10.3390/molecules30010100)
Supplement: Supplementary file 1 [file molecules-30-00100-s001.zip › molecules-3368273-supplementary.pdf]

Supplementary Information

Structure- and ligand-based virtual screening for identification of novel TRPV4 antagonists

Atefeh Saadabadi [1,2,3,*], Linda Wilkman [1,2], Marja Rantanen [4], Ari-Pekka Koivisto [4] and Outi M. H. Salo-Ahen [1,2,*]

[1] Structural Bioinformatics Laboratory, Faculty of Science and Engineering, Åbo Akademi University, Tykistökatu 6, 20520 Turku, Finland.

[2] Pharmaceutical Sciences Laboratory, Faculty of Science and Engineering, Åbo Akademi University, Tykistökatu 6, 20520 Turku, Finland.

[3] Laboratory of Molecular Science and Engineering, Faculty of Science and Engineering, Åbo Akademi University, Henrikinkatu 2, 20500 Turku, Finland

[4] Pain Therapy Area, Orion Pharma, Tengströminkatu 8, 20360 Turku, Finland

*Corresponding author: atefeh.saadabadi@abo.fi; outi.salo-ahen@abo.fi

This document includes:

Figure S1. Structures of TRPV4 agonists and antagonists 3

Table S1. The cryo-EM structures of TRPV4 in the Protein Data Bank 4

Clustal O (1.2.4) multiple sequence alignment 5

Figure S2. The Ramachandran plots 6

Figure S3. RMSD evolution of the hTRPV4 model during a 300-ns MD simulation 7

Figure S4. Multiple-sequence alignment 8

Figure S5. Identifying a ligand binding site in the hTRPV4 model 9

Figure S6. The SiteMap result for the pocket that was selected for structure-based virtual screening 10

Figure S7. Superposition of the TM domains of the hTRPV4 model and the experimental structure of hTRPV4. 11

Figure S8. The conformation of the SiteMap-predicted binding site 12

Figure S9. Chemical structures of the top-ranked compounds from the structure-based virtual screening by molecular docking. 13

Figure S10. The docked poses of the selected virtual hit compounds in the predicted binding site of the hTRPV4 model 14

Figure S11. The pharmacophore used for virtual screening of TRPV4 inhibitors 15

Figure S12. IC50 curve for the virtual screening hits at hTRPV4. 16

Table S2. The molecular docking results of the natural stilbenoids in the hTRPV4 model 17

Figure S13. The docking poses of the natural stilbenoids and the known reference compounds 18

Figure S14. EC_{50} and IC_{50} curves for the stilbenoids at hTRPV4 19

Table S3. Predicted binding free energies of the biologically active hTRPV4 hits 20

Figure S15. The MD simulation analysis of Z1213 in the 4α-PDD binding site of the hTRPV4 model 21

Molecular docking study using the cryo-EM structure of hTRPV4 22

Table S4. Predicted binding free energies of the structure-based virtual screening hits and the natural stilbenoids at the 4αPDD binding site in the hTRPV4 model and the experimental hTRPV4 structure 23

Figure S16. The MD simulation system for TRPV4 model-ligand Z1213 complex. 24

References 25

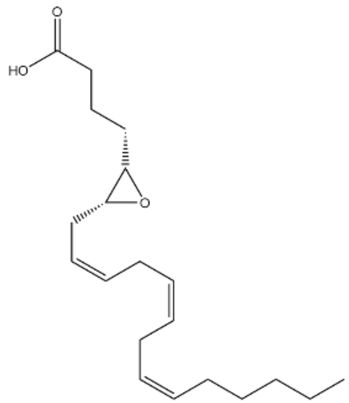

5′,6′-EET

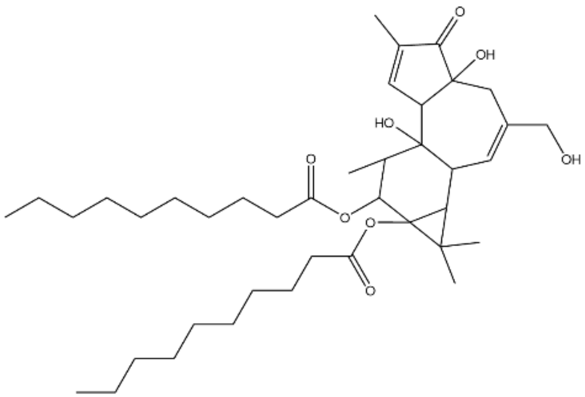

4α-PDD

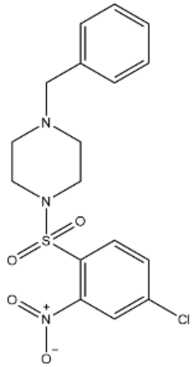

RN-1774

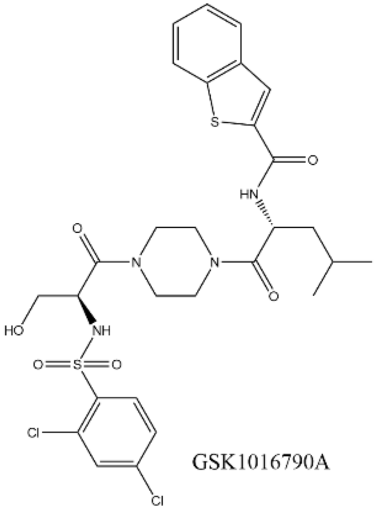

GSK1016790A

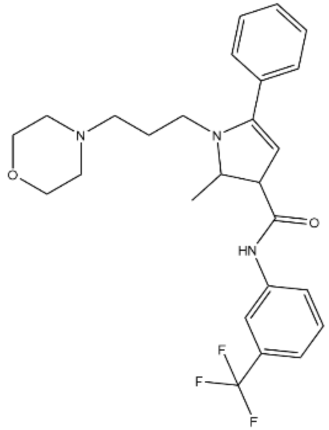

HC-067047

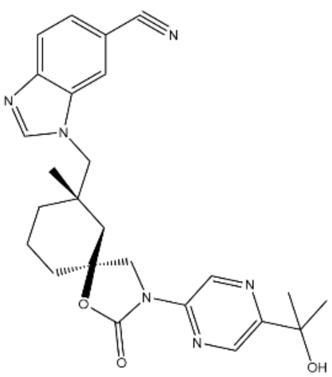

GSK2798745

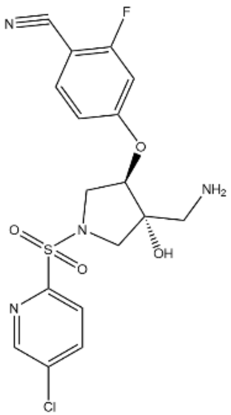

GSK3527497

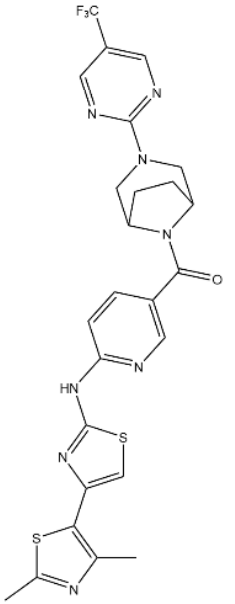

antagonist A2

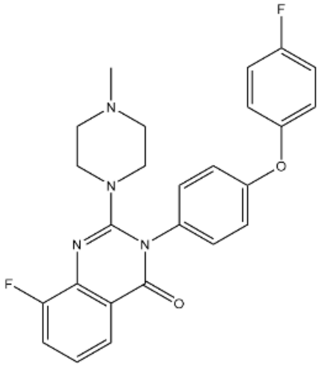

Agonist1

Figure S1. Structures of TRPV4 agonists and antagonists.

Table S1. The cryo-EM structures of TRPV4 in the Protein Data Bank (PDB) (as of 25th September 2024)

Structure PDB ID	Year	Organism	Small molecular ligand in the complex	Ligand binding site	State	Resolution (Å)
6BBJ[1]	2018	*Xenopus tropicalis*	-	-	closed	3.8
7AA5[2]	2021	*Homo sapiens*	4α-PDD (agonist)	Density at the base of the VSLD	open	4.18
8T1B[3]	2023	*Homo sapiens*	-		closed	3
8T1D[3]	2023	*Homo sapiens*	4α-PDD (agonist)	at the base of the VSLD	open	3.35
8T1E[3]	2023	*Homo sapiens*	4α-PDD (agonist)	Density at the base of the VSLD	closed	2.77
8T1F[3]	2023	*Homo sapiens*	HC067047 (antagonist)	at the base of the VSLD	partially closed	3.49
8FC7[4]	2023	*Homo sapiens*	GSK2798745 (antagonist)	at the base of the VSLD	closed	3.3
8FC8[4]	2023	*Homo sapiens*	GSK1016790A (agonist) 4	at the base of the VSLD	open	3.47
8FC9[4]	2023	*Homo sapiens*	-	-	intermediate*	3.75
8FCA[4]	2023	*Homo sapiens*	4α-PDD (agonist)	at the base of the VSLD	open	3.6
8FCB[4]	2023	*Homo sapiens*	GSK1016790A (agonist) 4	at the base of the VSLD	open	3.52
8J1B[5]	2023	*Mus musculus*	GSK1016790A (agonist)<br>ruthenium red (antagonist)	- at the base of the VSLD<br>- at the extracellular entrance of the selectivity filter	open	3.72
8J1D[5]	2023	*Mus musculus*	-	-	closed	3.59
8J1F[5]	2023	*Mus musculus*	GSK1016790A (agonist)	at the base of the VSLD	open	3.62
8J1H[5]	2023	*Mus musculus*	Agonist1<br>ruthenium red (antagonist)	at the base of the VSLD<br>-	closed	3.88
8JKM[5]	2023	*Mus musculus*	RN-1747 (agonist)	-	closed	3.98
8JU5[6]	2024	*Homo sapiens*	GSK3527497 (antagonist)	at the base of the VSLD	closed	3.74
8JU6[6]	2024	*Homo sapiens*	GSK2798745 (antagonist)	at the base of the VSLD	closed	3.45
8JVI[6]	2024	*Homo sapiens*	Antagonist A2	at the base of the VSLD	closed	3.21
8JVJ[6]	2024	*Homo sapiens*	Antagonist A2	at the base of the VSLD	closed	3.44

* Lower gate is between open and closed states: the distance between M718 residues of opposite subunits is 6.9 Å (open – 8.3 Å and closed state – 4.9 Å).

Clustal O (1.2.4) multiple sequence alignment

```
sp|Q9HBA0|hTRPV4      MADSSEGPRAGPGEVAELPGDESGTPGGEAFPLSSLANLFEGEDGSLSPSPADASRPAGP   60
tr|F7BWY7|xTRPV4      MADPSHLLKHNASVDIDDSQGDDGSNHNDSFPLSSLANLFENEESSAPN—EGVRSPQVP    58
6BBJ_xTRPV4           MADPSHLLKHNASVDIDDSQGDDGSNHNDSFPLSSLANLFENEESSAPN—EGVRSPQVP    58
                      *** *.  : . .   :   .:.*:  .::**********.*:.*      ..  *  *

sp|Q9HBA0|hTRPV4      GDGRPNLRMKFQGAFRKGVPNPIDLLESTLYESSVVPGPKKAPMDSLFDYGTYRHHSSDN   120
tr|F7BWY7|xTRPV4      GDNKQNLRIRFQGPFRKGISNPMDLLESTIYESS---APKKAPMDSLFGYETYHHHPTEN   115
6BBJ_xTRPV4           GDNKQNLRIRFQGPFRKGISNPMDLLESTIYESS---APKKAPMDSLFGYETYHHHPTEN   115
                      **.: ***::*** ****: **:******:****    .**********.* **:** ::*

sp|Q9HBA0|hTRPV4      KRWRKKII-EKQPQSPKAPAPQPPPILKVFNRPILFDIVSRGSTADLDGLLPFLLTHKKR   179
tr|F7BWY7|xTRPV4      RRKRKKILLEKENLNSQAPSPDPPPVIKMFNRHMLFDIVSRGSTAELEGFLPFLLAQKKR   175
6BBJ_xTRPV4           RRKRKKILLEKENLNSQAPSPDPPPVIKMFNRHMLFDIVSRGSTAELEGFLPFLLAQKKR   175
                      :* ****: **:   . :**:*:***::*:*** :***********:*:*:*****::***

sp|Q9HBA0|hTRPV4      LTDEEFREPSTGKTCLPKALLNLSNGRNDTIPVLLDIAERTGNMREFINSPFRDIYYRGQ   239
tr|F7BWY7|xTRPV4      LTDEEFREASTGKTCLTKALMNLNGGKNDTIPMLIDIAEKTGNLREFINSPFRDVYYRGQ   235
6BBJ_xTRPV4           LTDEEFREASTGKTCLTKALMNLNGGKNDTIPMLIDIAEKTGNLREFINSPFRDVYYRGQ   235
                      ******** ******* ***:**..*:*****:*:****:***:**********:*****

sp|Q9HBA0|hTRPV4      TALHIAIERRCKHYVELLVAQGADVHAQARGRFFQPKDEGGYFYFGELPLSLAACTNQPH   299
tr|F7BWY7|xTRPV4      TALHIAIERRCKHYVELLVEKGADVHAQARGRFFQPKDEGGYFYFGELPLSLAACTNQPD   295
6BBJ_xTRPV4           TALHIAIERRCKHYVELLVEKGADVHAQARGRFFQPKDEGGYFYFGELPLSLAACTNQPD   295
                      ******************* :***************************************.

sp|Q9HBA0|hTRPV4      IVNYLTENPHKKADMRRQDSRGNTVLHALVAIADNTRENTKFVTKMYDLLLLKCARLFPD   359
tr|F7BWY7|xTRPV4      IVHYLTENAHKKADIRRQDSRGNTVLHALVAIADNTRENTKFVTKVYDLLVIKCVKLYPD   355
6BBJ_xTRPV4           IVHYLTENAHKKADIRRQDSRGNTVLHALVAIADNTRENTKFVTKVYDLLVIKCVKLYPD   355
                      **:***** *****:******************************:****::**.:*:**

sp|Q9HBA0|hTRPV4      SNLEAVLNNDGLSPLMMAAKTGKIGIFQHIIRREVTDEDTRHLSRKFKDWAYGPVYSSLY   419
tr|F7BWY7|xTRPV4      SSLEAIFNNDSMSPLMMAAKLGKIGIFQHIIRLEIKDEEARHLSRKFRDWAYGPVYSSLY   415
6BBJ_xTRPV4           SSLEAIFNNDSMSPLMMAAKLGKIGIFQHIIRLEIKDEEARHLSRKFRDWAYGPVYSSLY   415
                      *.***::***.:******** *********** *:.**::*******:************

sp|Q9HBA0|hTRPV4      DLSSLDTCGEEASVLEILVYNSKIENRHEMLAVEPINELLRDKWRKFGAVSFYINVVSYL   479
tr|F7BWY7|xTRPV4      DLSMLDTCGEEVSVLEILVYNSKVENRHEMLAVEPINELLRDKWQKFGAVSFYISVVSYL   475
6BBJ_xTRPV4           DLSMLDTCGEEVSVLEILVYNSKVENRHEMLAVEPINELLRDKWQKFGAVSFYISVVSYL   475
                      *** *******.***********:********************:*********.*****

sp|Q9HBA0|hTRPV4      CAMVIFTLTAYYQPLEGTPPYPYRTTVDYLRLAGEVITLFTGVLFFFTNIKDLFMKKCPG   539
tr|F7BWY7|xTRPV4      IAMIIFTLIAYYRPMDGTPPYPYRTTMDYMRLAGEIVTLLTGVVFFITNIKDLFMKKCPG   535
6BBJ_xTRPV4           IAMIIFTLIAYYRPMDGTPPYPYRTTMDYMRLAGEIVTLLTGVVFFITNIKDLFMKKCPG   535
                       **:**** ***:*::*********:**:*****::**:***:**:*************

sp|Q9HBA0|hTRPV4      VNSLFIDGSFQLLYFIYSVLVIVSAALYLAGIEAYLAVMVFALVLGWMNALYFTRGLKLT   599
tr|F7BWY7|xTRPV4      VNSLFIDGSFQLLYFIYSVLVIITAVLYLVGIESYLAVMVFALVLGWMNALYFTRGLKLT   595
6BBJ_xTRPV4           VNSLFIDGSFQLLYFIYSVLVIITAVLYLVGIESYLAVMVFALVLGWMNALYFTRGLKLT   595
                      **********************::*.***.***:**************************

sp|Q9HBA0|hTRPV4      GTYSIMIQKILFKDLFRFLLVYLLFMIGYASALVSLLNPCANMKVCNEDQTNCTVPTYPS   659
tr|F7BWY7|xTRPV4      GTYSIMLQKILFKDLFRFLLVYLLFMIGYASALVSLLNPCTSQESCIETSSNCTVPEYPS   655
6BBJ_xTRPV4           GTYSIMLQKILFKDLFRFLLVYLLFMIGYASALVSLLNPCTSQESCIETSSNCTVPEYPS   655
                      ******:*********************************:.  : * *  .:***** ***

sp|Q9HBA0|hTRPV4      CRDSETFSTFLLDLFKLTIGMGDLEMLSSTKYPVVFIILLVTYIILTFVLLLNMLIALMG   719
tr|F7BWY7|xTRPV4      CRDSSTFSKFLLDLFKLTIGMGDLEMINSAKYPAVFIILLVTYIILTFVLLLNMLIALMG   715
6BBJ_xTRPV4           CRDSSTFSKFLLDLFKLTIGMGDLEMINSAKYPAVFIILLVTYIILTFVLLLNMLIALMG   715
                      ****.***.*****************:.*:***.**************************

sp|Q9HBA0|hTRPV4      ETVGQVSKESKHIWKLQWATTILDIERSFPVFLRKAFRSGEMVTVGKSSDGTPDRRWCFR   779
tr|F7BWY7|xTRPV4      ETVGQVSKESKQIWKLQWATTILDIERSFPVCMRKAFRSGEMVTVGKNLDGTPDRRWCFR   775
6BBJ_xTRPV4           ETVGQVSKESKQIWKLQWATTILDIERSFPVCMRKAFRSGEMVTVGKNLDGTPDRRWCFR   775
                      ***********:******************* :**************. **********

sp|Q9HBA0|hTRPV4      VDEVNWSHWNQNLGIINEDPGKNETYQYYGFSHTVGRLRRDRWSSVVPRVVELNKNSN-P   838
tr|F7BWY7|xTRPV4      VDEVNWSHWNQNLGIINEDPGRNDGYQYYGFSQTVGRLRRDRWSVVVPRVVELNKAPQHS   835
6BBJ_xTRPV4           VDEVNWSHWNQNLGIINEDPGRNDGYQYYGFSQTVGRLRRDRWSVVVPRVVELNKAPQHS   835
                      *********************:*: *******:*********** *********   :

sp|Q9HBA0|hTRPV4      DEVVVPLDSMGNPRCDGHQQGYPRKWRTDDAPL                    871
tr|F7BWY7|xTRPV4      DDVVVPLGNIPQVQTYSQRQENAQNWKKDETHI                    868
6BBJ_xTRPV4           DDVVVPLGNIPQVQTYSQRQENAQNWKKDETHI                    868
                      *:*****..: : :  .::*   ::*:.*:: :
```

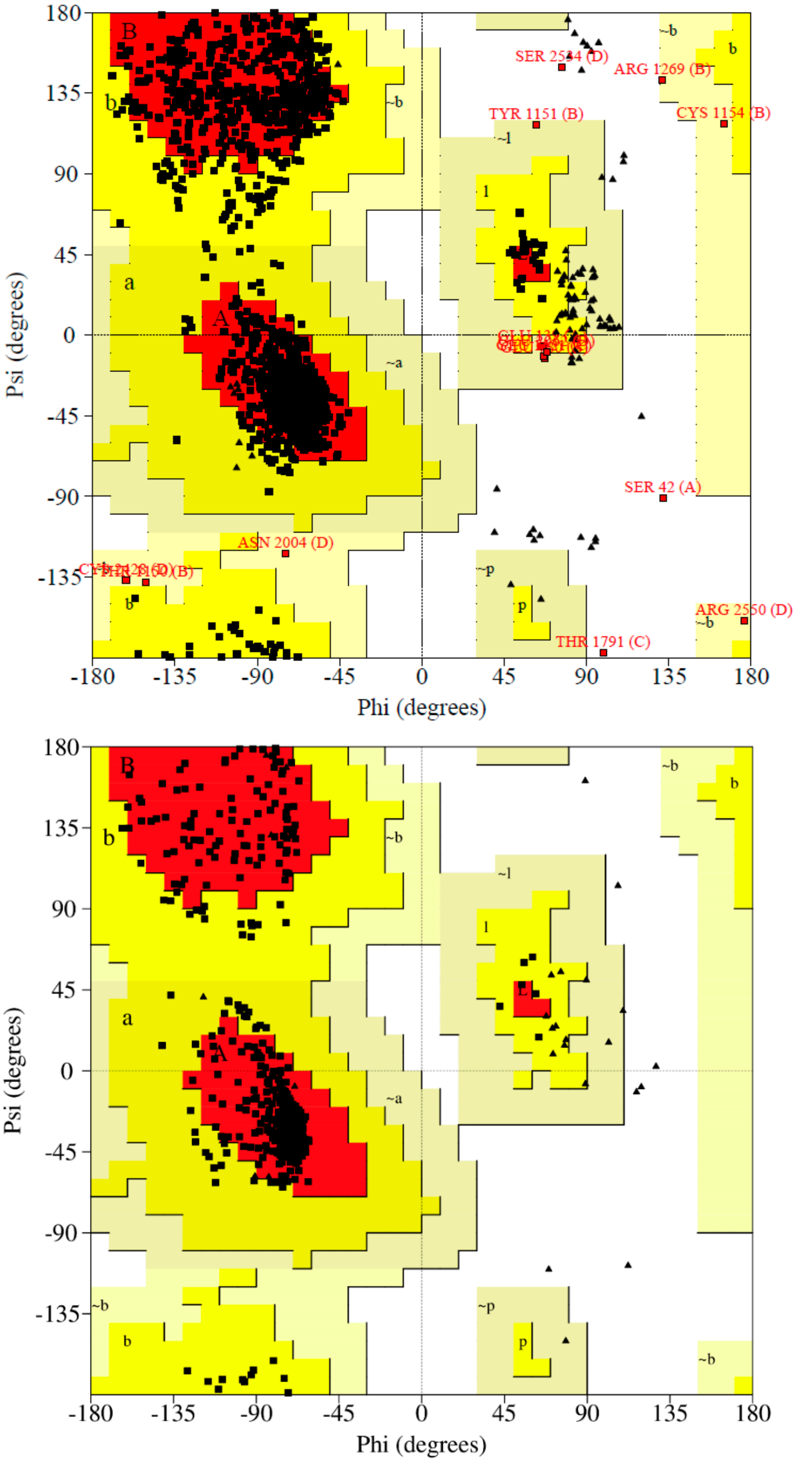

Figure S2. The Ramachandran plots for the human TRPV4 model (after protein preparation with Maestro) (top) and the template cryo-EM structure of *Xenopus tropicalis* TRPV4 (PDB ID: 6BBJ) (bottom). Sequence numbering in the hTRPV4 model: residue number 1 corresponds to res. 148 in the human TRPV4 sequence. Every chain contains residues 148-788 (numbering in the model – A: 1-641, B: 642-1282, C: 1283-1923, D: 1924-2564). Color code for the plot regions: red – most favoured; yellow – additional allowed; light yellow – generously allowed; white - disallowed.

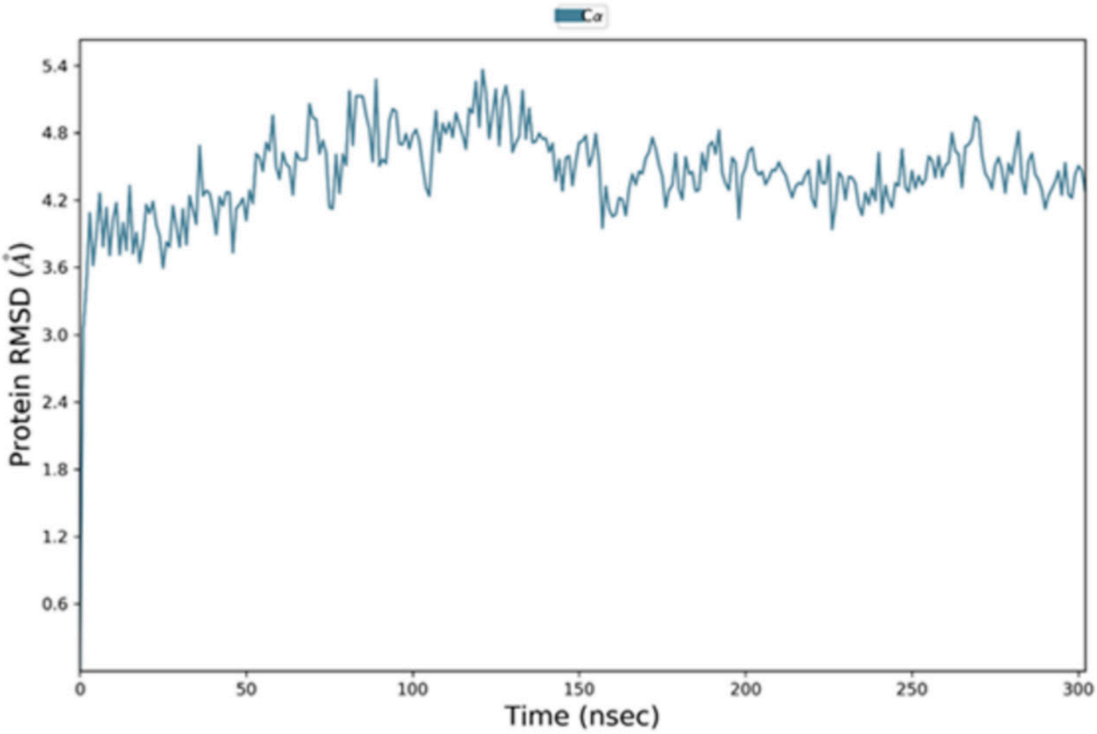

Figure S3. RMSD evolution of the hTRPV4 model during a 300-ns MD simulation.

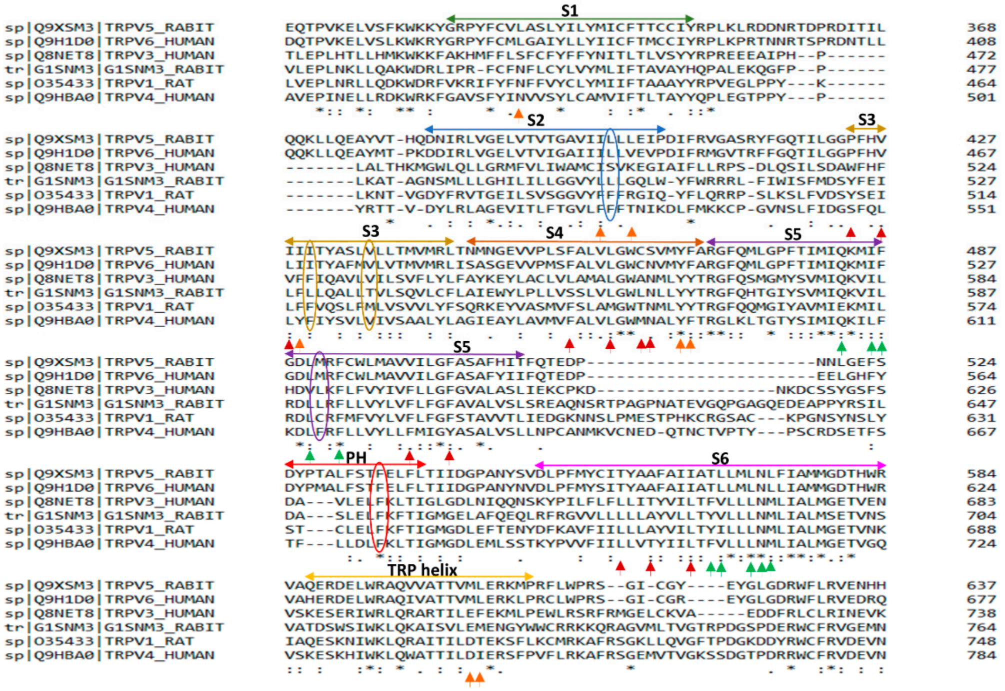

Figure S4. Multiple-sequence alignment of S1-S6, pore helix (PH) and TRP helix between rat TRPV1, rabbit TRPV2, hTRPV3, hTRPV4, rabbit TRPV5 and hTRPV6. The hTRPV4 residues interacting with some of the virtual screening hits are circled alongside with the corresponding residues in the other TRPV family members. Moreover, interacting residues of representative ligands binding to the VBS, VSLD and CBD binding sites in TRPVs are marked with colourful arrows: capsaicin in VBS (red), GSK2798745 in VSLD (orange); CBD (green). Conservation code: (*) identical residues, (:) conserved substitutions, (.) semi-conserved substitutions.

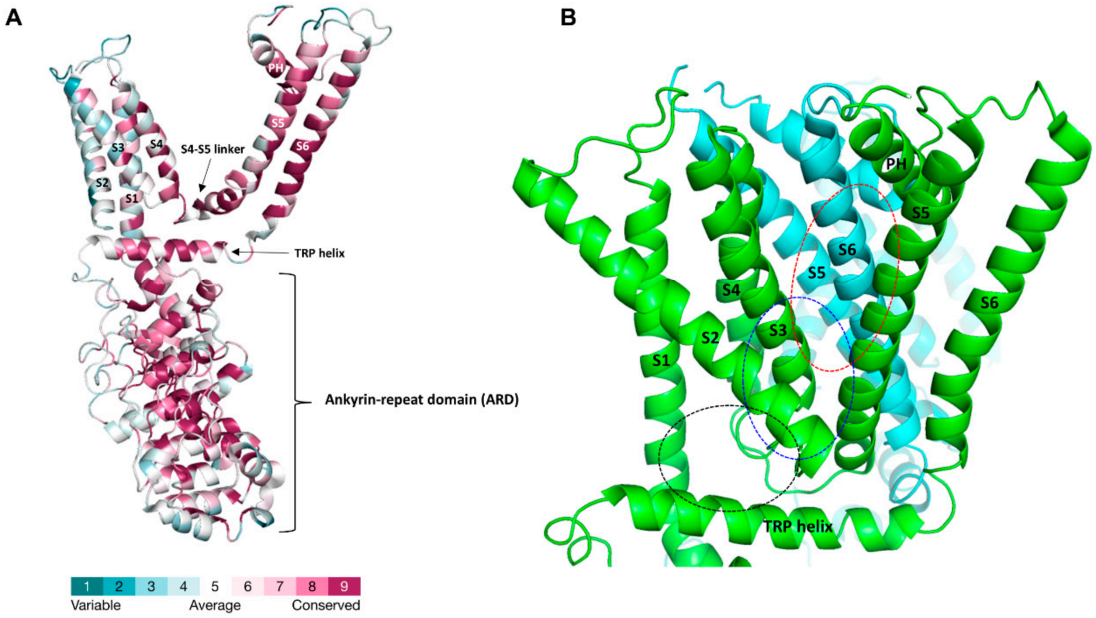

Figure S5. Identifying a ligand binding site in the hTRPV4 model. (A) The evolutionary conservation of amino acids in hTRPV4 (PDB ID: 7AA5, chain A) as predicted with the ConSurf webserver. The conservation level is colour-mapped on the hTRPV4 subunit. (B) The TMD conformation in the hTRPV4 model (two adjacent subunits are shown: green and cyan cartoon representation). The location of VSLD, VBS and the predicted site by SiteMap are presented in black, blue and red circles, respectively.

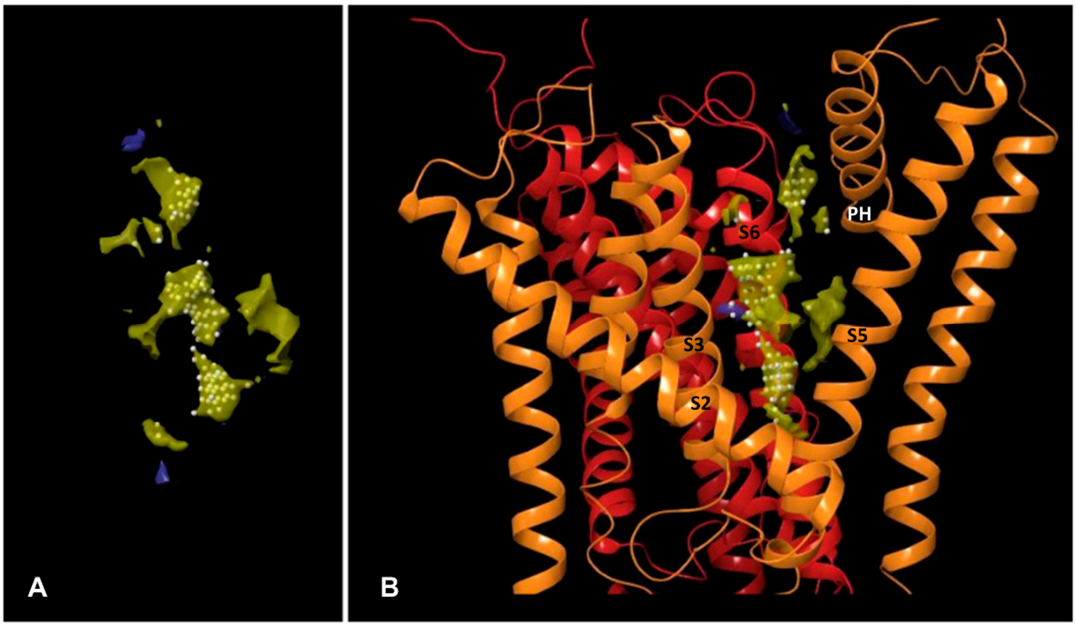

Figure S6. The SiteMap result for the pocket that was selected for structure-based virtual screening. (A) The binding site properties: hydrophobic region – yellow; hydrogen bond donor – purple; site points (that mark the extend of the pocket) – white spheres. (B) The binding site is located between the S3, S5 and pore helixes from one subunit (orange color) and the S6 helix from the adjacent subunit (red color)

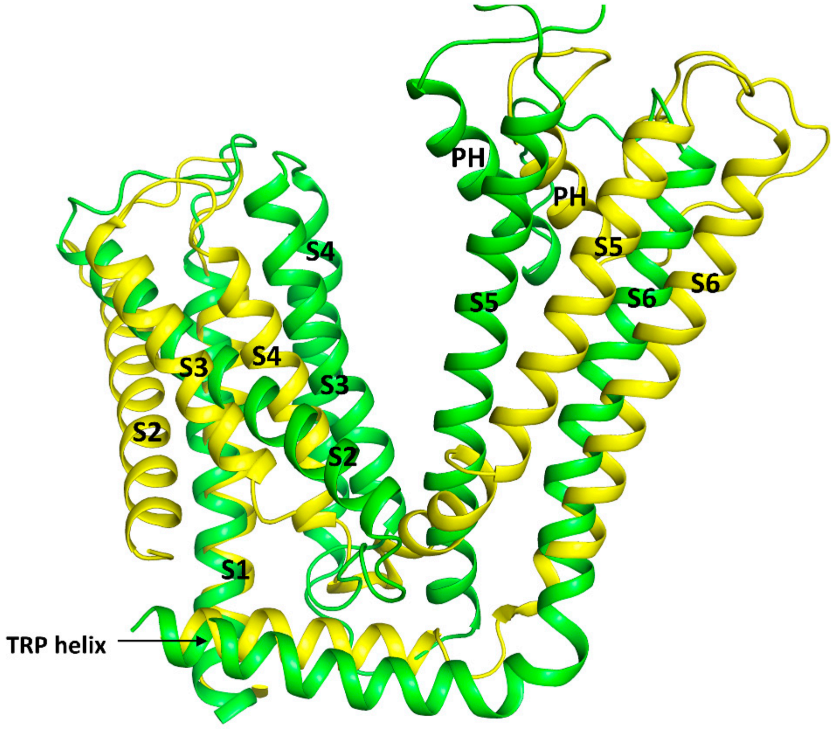

Figure S7. Superposition of the TM domains of the hTRPV4 model (green cartoon) and the experimental structure of hTRPV4 (PDB ID: 7AA5; yellow cartoon). The TM domains are labeled.

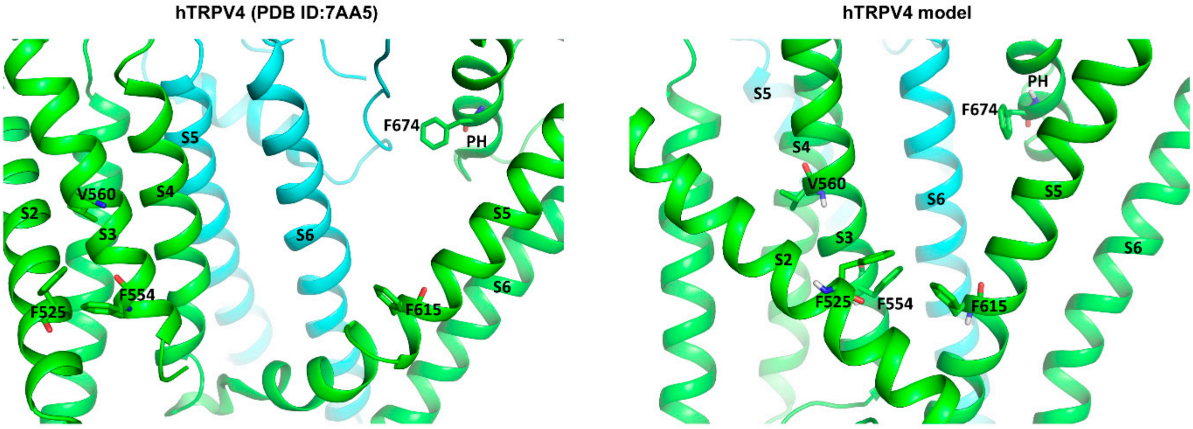

Figure S8. The conformation of the SiteMap-predicted binding site in the hTRPV4 model (right) and the experimental structure of hTRPV4 (left). Two adjacent subunits of the protein are shown in green and cyan cartoon representation. The key residues are shown as sticks and the TM domains are labelled. Non-polar hydrogen atoms are omitted for clarity.

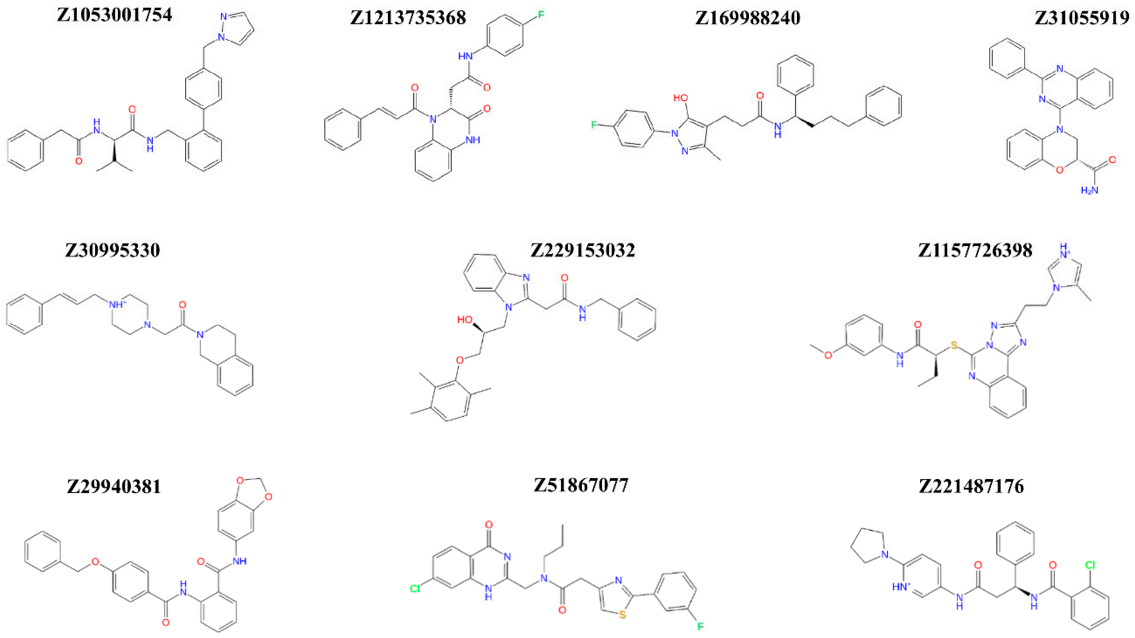

Figure S9. Chemical structures of the top-ranked compounds from the structure-based virtual screening by molecular docking.

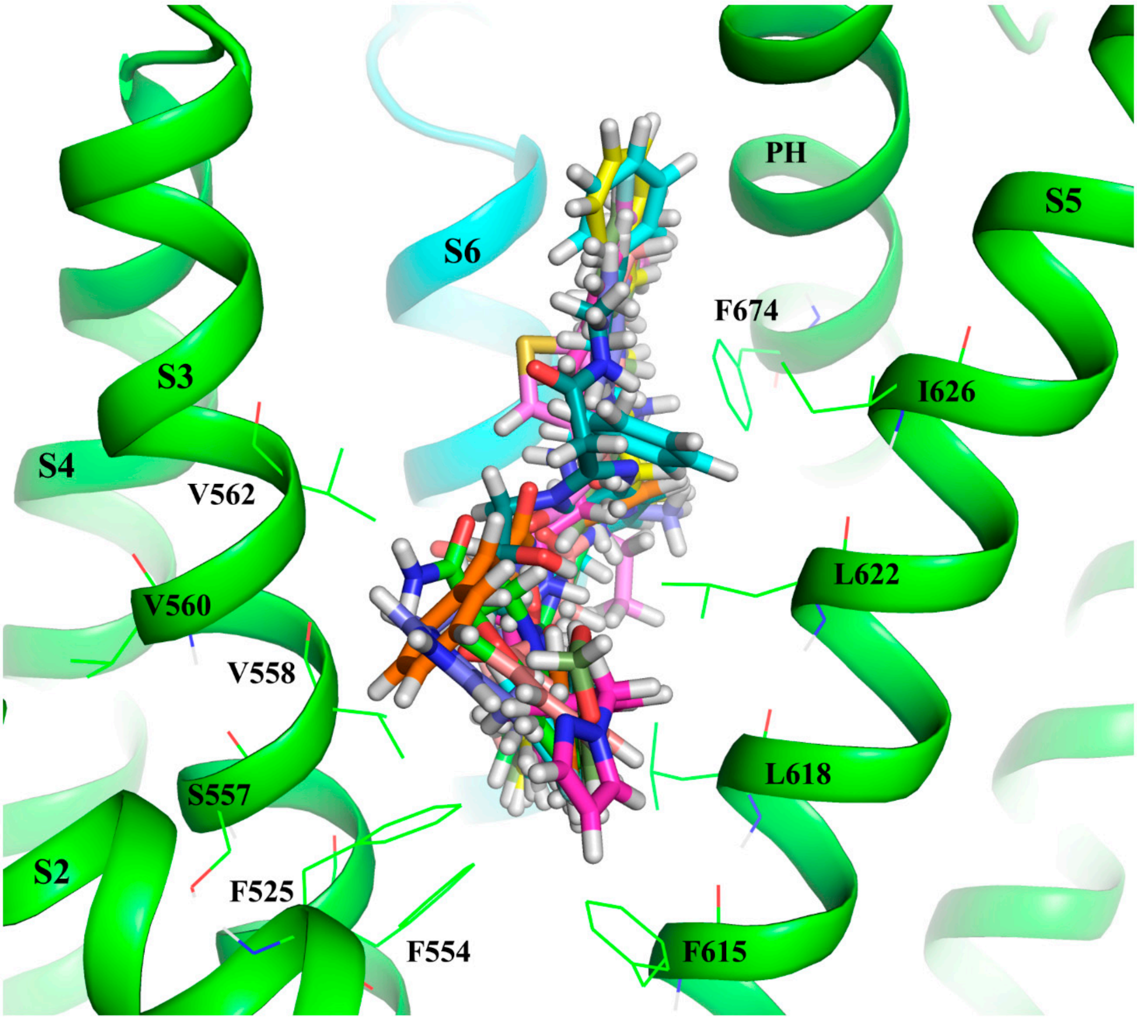

Figure S10. The docked poses of the selected virtual hit compounds in the predicted binding site of the hTRPV4 model. The hTRPV4 model is represented in cartoon (chain A – green; chain D – blue) and the compounds are shown in sticks. Atom color code: carbon – different color depending on the molecule; nitrogen - blue; oxygen - red; fluorine - light blue; chlorine – green; sulfur – gold; hydrogen – white. The key residues (in lines) and the TM domains are labelled. Non-polar hydrogen atoms are omitted for clarity.

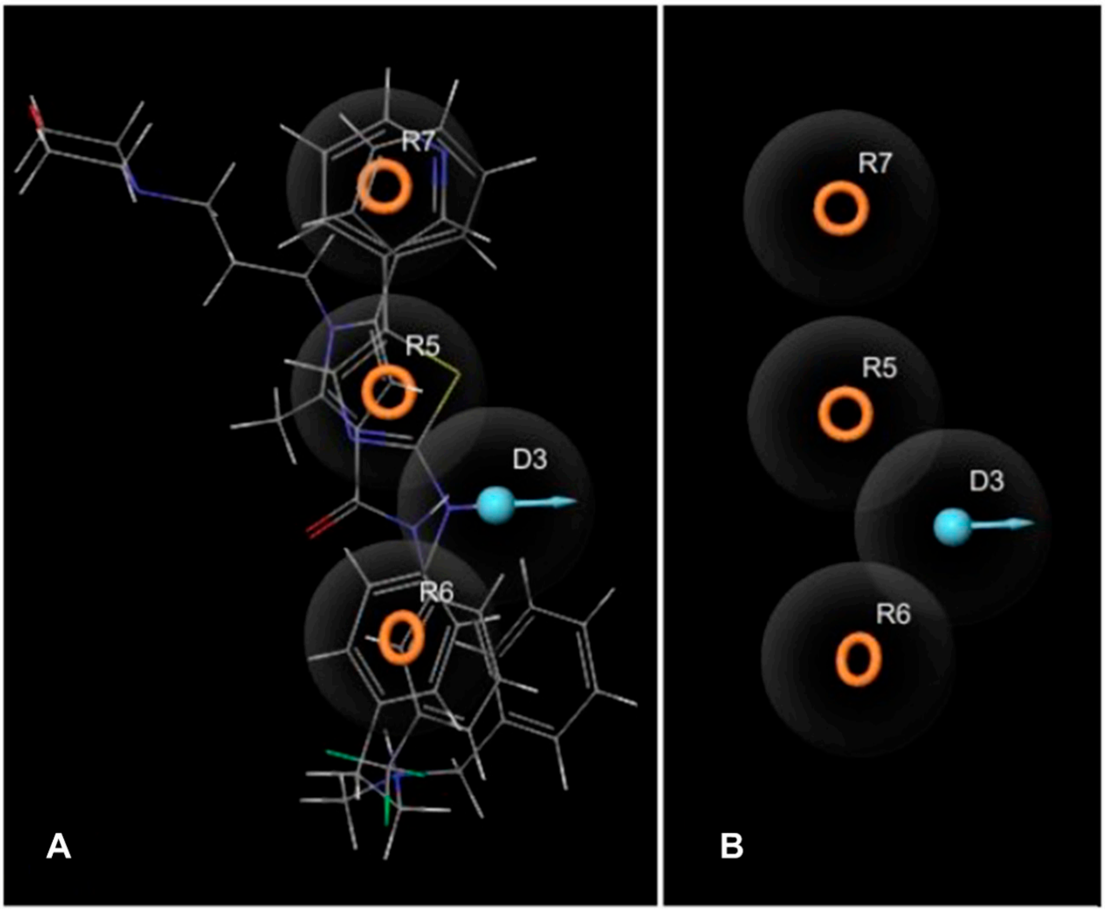

Figure S11. The pharmacophore used for virtual screening of TRPV4 inhibitors. (A) The alignment of GSK205 and HC-067047 with the best-ranked pharmacophore model. The settings used to generate the pharmacophore hypotheses were the following: the hypothesis should match at least one of the template molecules; the number of features: 4-7 but preferably at least 5; the criterion to reject redundant hypotheses (hypothesis difference): 0.50; scoring function for ranking the hypotheses: PhaseHypoScore; the number of hydrogen bond acceptors: 0-9; the number of hydrogen bond donors: 0-4. (B) Pharmacophore features: orange rings - aromatic rings (R); blue ball with an arrow - hydrogen bond donor (D).

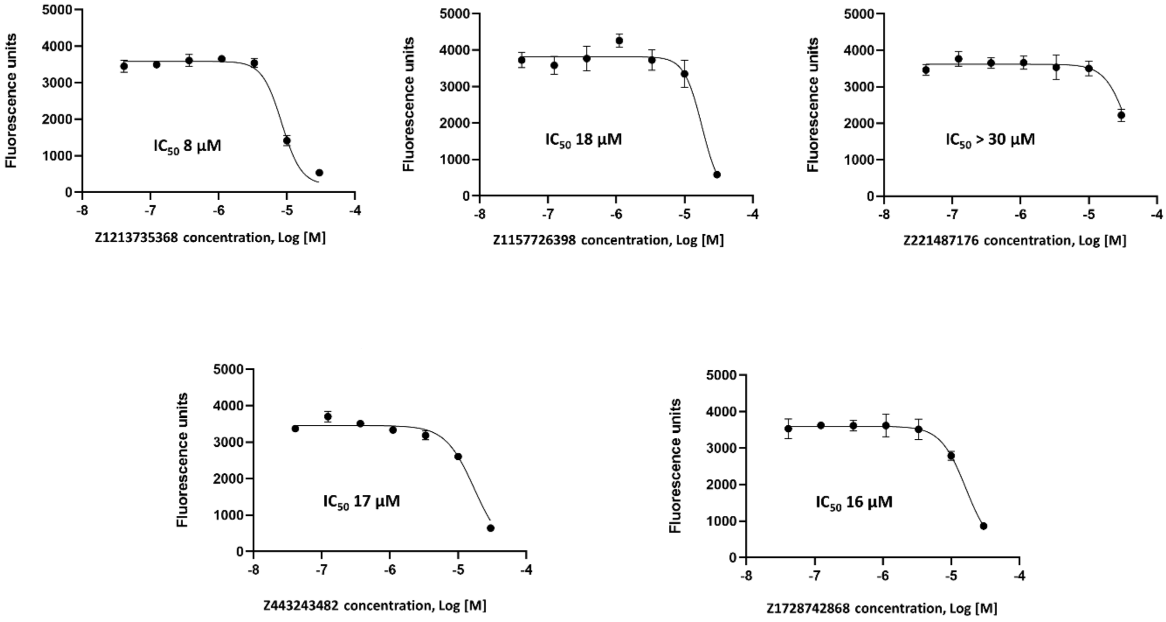

Figure S12. IC50 curve for the virtual screening hits at hTRPV4.

Table S2. The molecular docking results of the natural stilbenoids in the hTRPV4 model

Compounds	SiteMap-identified binding site		4α-PDD binding site	
	Glide XP GScore (kcal/mol)	Prime/ MM-GBSA ΔG-bind (kcal/mol)	Glide XP GScore (kcal/mol)	Prime/ MM-GBSA ΔG-bind (kcal/mol)
**Resveratrol**	-6.29	-33.66	-5.97	-38.69
**Pinosylvin**	-6.41	-34.85	-4.25	-43.84
**PsMME**	-4.71	-33.18	-5.32	-49.07
**Isorhapontin**	-5.64	-35.57	-8.01	-53.96
**Astringin**	-8.07	-41.58	-8.81	-53.30
**4α-PDD (reference)**	n.d.	n.d.	-8.10	-66.91

n.d. – not determined at this site

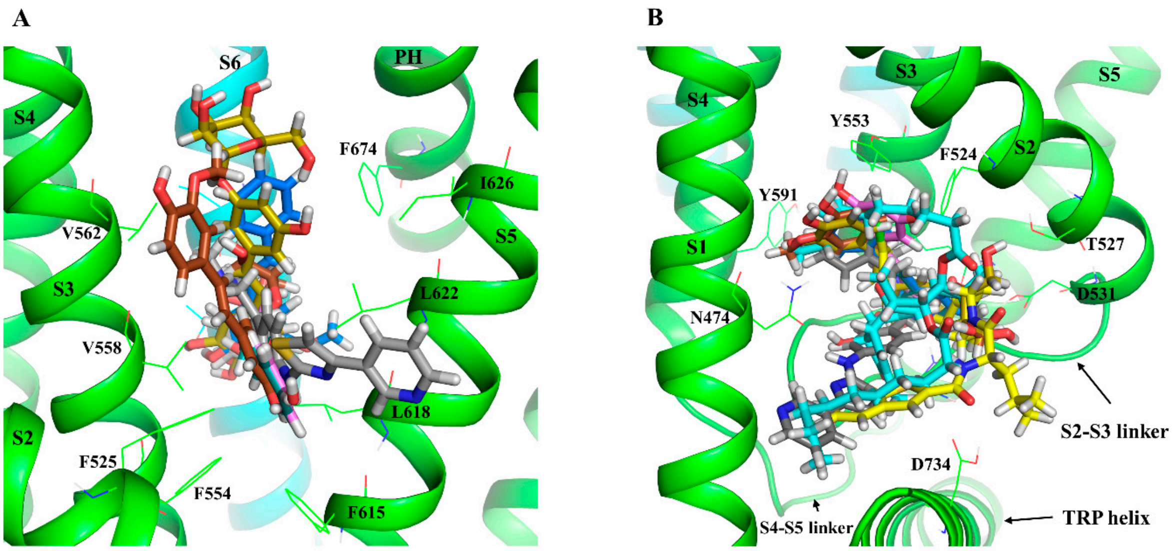

Figure S13. The docking poses of the natural stilbenoids and the known reference compounds at (A) the SiteMap predicted site and at (B) the 4α-PDD binding site reported by Botte et al.[1] of the hTRPV4 model. The hTRPV4 model is represented in cartoon (subunits colored differently) and compounds pinosylvin (teal), PsMME (blue), resveratrol (violet), astringin (olive), isorhapontin (brown), GSK205 (gray), GSK1016790A (yellow), and 4α-PDD (cyan) in sticks. Atom color code: carbon – according to the compound; nitrogen – blue; oxygen – red; sulfur – yellow; chlorine – green; hydrogen – white. The key residues (in lines) and the TM domains are labelled.

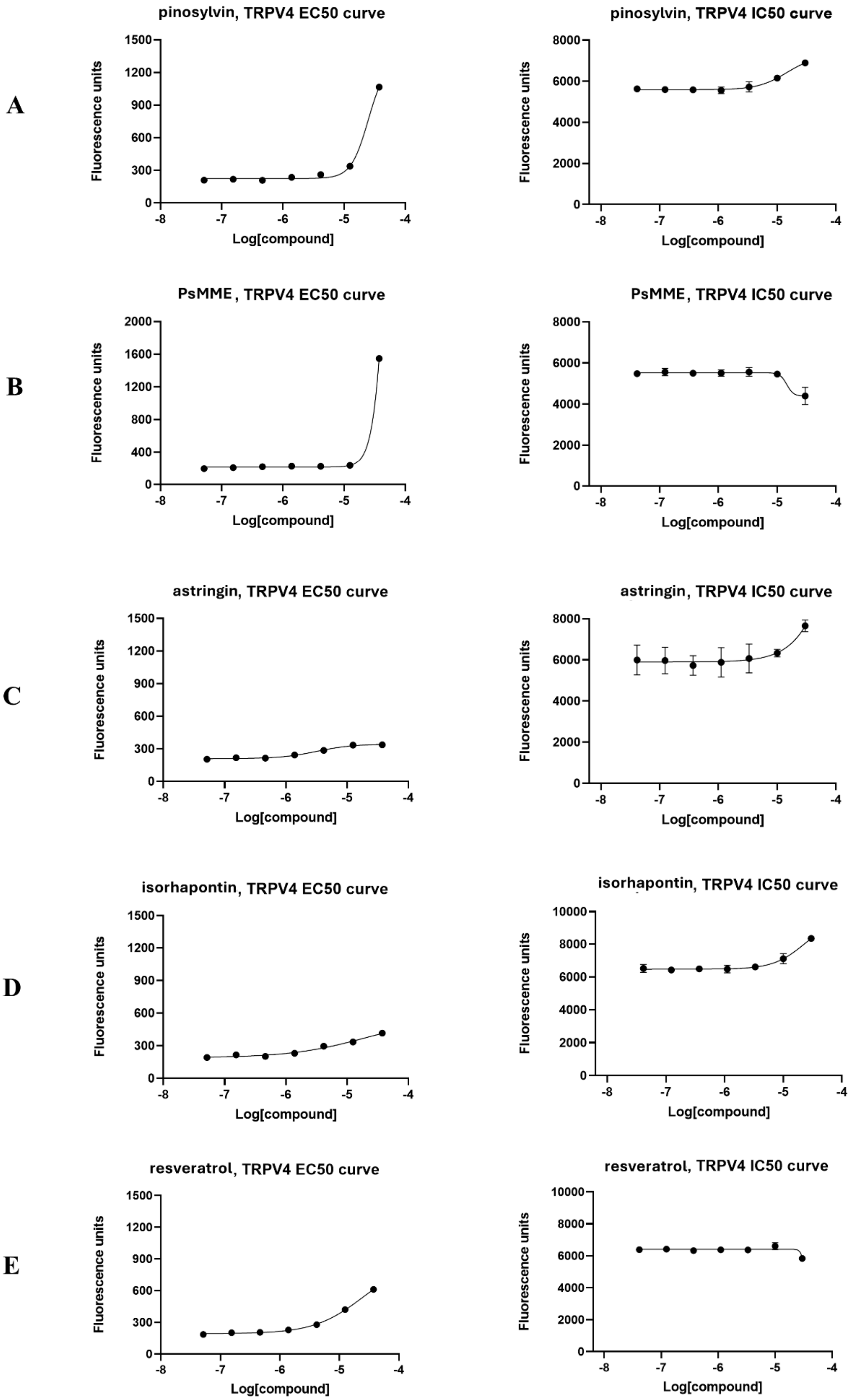

Figure S14. EC_{50} and IC_{50} curves for the stilbenoids at hTRPV4: pinosilvin (A), PsMME (B), astringin (C), isorhapontin (D), and resveratrol (E).

Table S3. Predicted binding free energies of the biologically active hTRPV4 hits from the structural-based and ligand-based virtual screening docked with Glide and GOLD at two putative binding sites in the ***hTRPV4 model***.

Compounds	**MM-GBSA ΔG-bind (kcal/mol) 4α-PDD binding site at VSLD**		**MM-GBSA ΔG-bind (kcal/mol) SiteMap-predicted binding site**	
	Glide	GOLD	Glide	GOLD
**Z2214**[a]	-68.02	-64.55	n.d.[b]	-60.48
**Z1213**[a]	-60.58	-49.10	-56.91	n.d.
**Z1157**[a]	-63.49	-65.99	n.d.	-61.29
**Z1728**[c]	-55.27	-56.81	-66.43	-66.59
**Z4432**[c]	-46.33	-51.08	-60.42	-62.10
**GSK205**[d]	-67.06	-69.06	-83.54	-82.33
**HC-067047**[d]	-61.08	-65.14	-78.66	-81.71
**4α-PDD**[e]	-64.78	-58.30	n.d.	n.d.

[a] hit from the structural-based virtual screening; [b] not determined; [c] hit from the ligand-based virtual screening. [d] a template molecule for the pharmacophore model; [e] the reference molecule

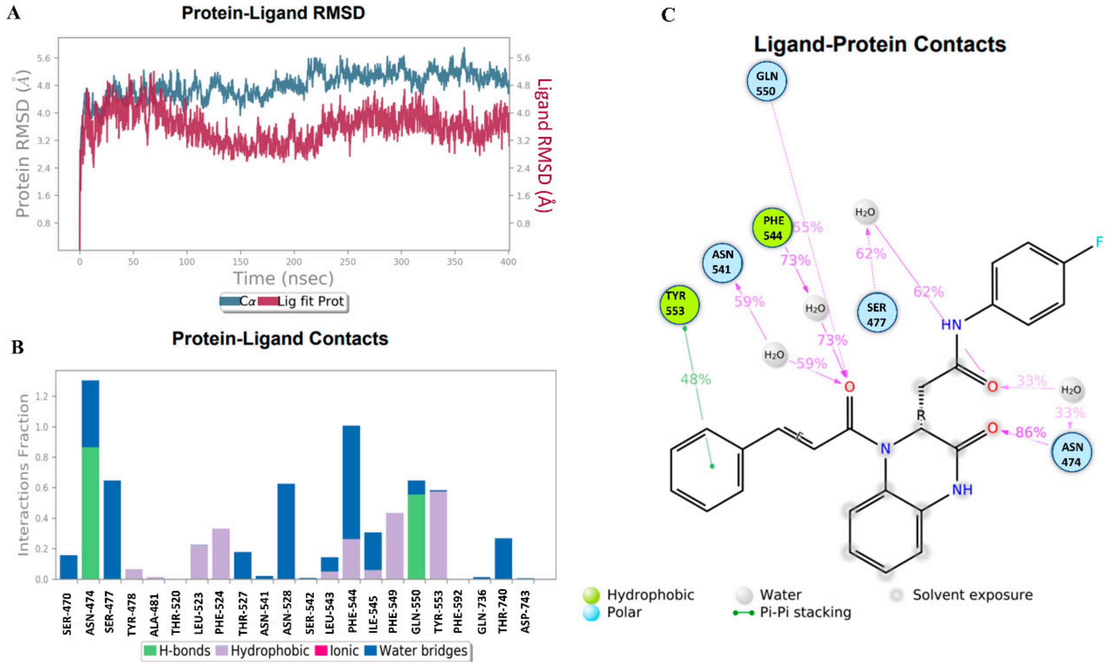

Figure S15. The MD simulation analysis of Z1213 in the 4α-PDD binding site of the ***hTRPV4 model***. (A) A RMSD plot for the hTRPV4 model in complex with the ligand during a 400-ns MD simulation. (B) The interactions fraction between the specific residues and the ligand (1.0 = interaction is maintained 100% of the simulation time; >1.0 = residue can form multiple interactions with the ligand). (C) The percentage of occupancy of the interactions that occurred during the simulation with the different functional groups of the ligand.

Molecular docking study using the cryo-EM structure of hTRPV4

The structure-based VS hits (compounds Z1213, Z1157, Z2214) and the natural stilbenoids were docked in the 4α-PDD binding site at the VSLD.[1] The docking site at the VSLD of the hTRPV4 (PDB ID:7AA5) was defined using the Receptor Grid Generation tool, centered on residues N474, Y591, R594, Y553, Y556, and S747. The ligand diameter midpoint box was set to 10 Å x 10 Å x 10 Å and the length of ligands to be docked was limited to 15 Å. Up to five poses per ligand were generated and the best pose for each ligand was selected for calculating the binding free energy with the Prime/MM-GBSA module of Maestro (Schrödinger Releases 2023-2; Prime, Schrödinger, LLC, New York, NY, 2020) using the VSGB solvation model[2] and the OPLS4[3] force field, keeping all binding site residues fixed. Flexible sampling was done by minimization of the complex.

Table S4. Predicted binding free energies of the structure-based virtual screening hits and the natural stilbenoids at the 4αPDD binding site in the hTRPV4 model and the experimental hTRPV4 structure (PDB ID:7AA5).

Compounds	MM-GBSA ΔG-bind (kcal/mol) 4αPDD binding site at VSLD	
	**hTRPV4 model**	**hTRPV4 (PDB ID: 7AA5)**
**Z2214**	-68.02	-54.84
**Z1213**	-60.58	-49.12
**Z1157**	-63.49	-56.08
**Resveratrol**	-38.69	-42.52
**Pinosylvin**	-43.84	-35.28
**PsMME**	-49.07	-33.95
**Isorhapontin**	-53.96	-44.51
**Astringin**	-53.30	-46.71
**4αPDD**	-64.78	-97.74*

*4αPDD could not be docked with normal Glide into this structure, so we used the induced fit docking of Maestro (IFD-MD, Schrödinger, LLC, New York, NY, 2022).[4–6] N.B. Botte et al.[1] used the Glide EM docking[7] in their work.

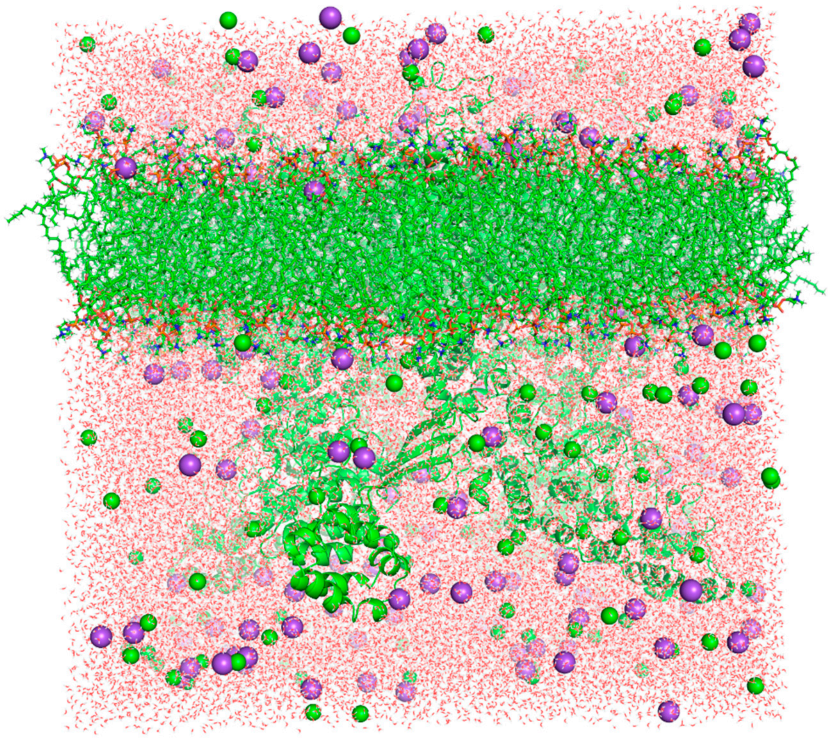

Figure S16. The MD simulation system for TRPV4 model-ligand Z1213 complex. TRPV4 ion channel, lipid membrane (POPC, 635 molecules), Na ions (237 molecules), Cl ions (20+237 molecules) and water molecules (TIP3P, 84959 molecules) are presented in green cartoon, sticks, purple spheres, green spheres and red-white lines, respectively.

References

1. Botte, M. *et al.* Cryo-EM structural studies of the agonist complexed human TRPV4 ion-channel reveals novel structural rearrangements resulting in an open-conformation. *bioRxiv* 2020.10.13.334797 (2020).

2. Li, J. *et al.* The VSGB 2.0 model: A next generation energy model for high resolution protein structure modeling. *Proteins: Structure, Function and Bioinformatics* **79**, (2011).

3. Lu, C. *et al.* OPLS4: Improving force field accuracy on challenging regimes of chemical space. *J Chem Theory Comput* **17**, 4291–4300 (2021).

4. Sherman, W., Beard, H. S. & Farid, R. Use of an induced fit receptor structure in virtual screening. *Chemical Biology and Drug Design* vol. 67 Preprint at https://doi.org/10.1111/j.1747-0285.2005.00327.x (2006).

5. Sherman, W., Day, T., Jacobson, M. P., Friesner, R. A. & Farid, R. Novel procedure for modeling ligand/receptor induced fit effects. *J Med Chem* **49**, (2006).

6. Farid, R., Day, T., Friesner, R. A. & Pearlstein, R. A. New insights about HERG blockade obtained from protein modeling, potential energy mapping, and docking studies. *Bioorg Med Chem* **14**, (2006).

7. Robertson, M. J., van Zundert, G. C. P., Borrelli, K. & Skiniotis, G. GemSpot: A Pipeline for Robust Modeling of Ligands into Cryo-EM Maps. *Structure* **28**, (2020).
